# COVID-19-related voice disorders: a scoping review

**DOI:** 10.1590/2317-1782/e20250243en

**Published:** 2026-05-08

**Authors:** Aline de Souza Silva, Rodrigo Dornelas, José Roberto Lapa e Silva

**Affiliations:** 1 Faculdade de Medicina, Universidade Federal do Rio de Janeiro – UFRJ - Rio de Janeiro (RJ), Brasil.

**Keywords:** COVID-19, Voice Disorders, Speech-Language Pathology, Coronavirus Infection, Voice Quality, Scoping Review

## Abstract

**Purpose:**

To map the available evidence on voice changes in non-intubated adults diagnosed with mild to moderate COVID-19.

**Research strategies:**

Scoping review conducted according to PRISMA-ScR guidelines, including studies published between 2019 and 2025. Systematic searches were performed in the MEDLINE (PubMed), EMBASE, LILACS, Scopus, Web of Science, and Cochrane Library databases and in grey literature sources (Google Scholar, MedRxiv, and ProQuest). Controlled descriptors and free terms related to COVID-19 and voice disorders were combined using Boolean operators.

**Selection criteria:**

The review included studies with adults (18–65 years) with a confirmed diagnosis of mild to moderate COVID-19 and excluded studies with individuals undergoing endotracheal intubation and with a previous history of voice disorders or respiratory comorbidities. The selection was performed by two independent reviewers.

**Data analysis:**

The data were extracted and analyzed descriptively and quantitatively, considering study characteristics, vocal assessment methods, and main outcomes.

**Results:**

Of the 35,497 records identified, 19 studies met the inclusion criteria. The most frequent voice disorders were dysphonia, hoarseness, reduction in maximum phonation time, and changes in acoustic measures such as jitter, shimmer, and harmonic-to-noise ratio. Associated symptoms included vocal fatigue, cough, dyspnea, and laryngeal discomfort, as well as a negative impact on voice-related quality of life.

**Conclusion:**

Mild to moderate COVID-19 can lead to clinically relevant vocal impairments, reinforcing the need for speech-language-hearing follow-up and research to support vocal assessment and rehabilitation protocols in the post-infection period.

## INTRODUCTION

COVID-19, caused by the SARS-CoV-2 virus, was declared a global pandemic in March 2020, affecting millions of people worldwide^(1)^. In addition to acute respiratory manifestations, the disease can trigger a cytokine storm, resulting in multisystemic damage, including the lungs, heart, and gastrointestinal tract^(2)^. Among the frequently reported symptoms are sensory alterations, such as hyposmia, anosmia, and dysgeusia, as well as respiratory manifestations that can directly impact voice production^(3)^.

Healthy voice production depends on the harmonious integration of the respiratory, phonatory, and resonance systems^(4)^. COVID-19 can compromise each of these systems through different mechanisms, such as laryngeal inflammation, peripheral neuropathies, respiratory muscle weakness, and postural changes resulting from chronic fatigue^(5,6)^. These can trigger dysphonia, defined as any difficulty or alteration in vocal emission that impairs natural voice production^(7)^.

Although COVID-19 has less clinical relevance compared to the early years of the pandemic, the study of vocal changes associated with the infection remains relevant. They can be part of the post-COVID syndrome (long COVID), recognized by the World Health Organization, posing challenges for rehabilitation and clinical follow-up^(8)^. Even mild vocal changes can have a significant functional impact, especially in individuals who depend on their voice for work, such as teachers, singers and communication professionals^(9,10)^. The scarcity of longitudinal studies investigating the duration, evolution, and therapeutic management of these changes highlights a gap in the literature, reinforcing the relevance of a scoping review^(11)^.

This review adopts the concept of vocal quality as the functional performance of the voice, assessed through auditory-perceptual, acoustic, and self-report measures^(7,12,13)^. The COVID-19 phenomenon is addressed in its full spectrum (acute, post-acute, and long COVID phases)^(8)^, considering its potentially impactful pathophysiological mechanisms on the voice, such as laryngeal inflammation and neuropathy^(5,6)^. The context is limited to the population of non-intubated adults with mild to moderate cases, allowing the isolation of the effects of viral infection from intubation damage and filling an important gap in the literature, which traditionally prioritizes the study of severe cases.

A preliminary search in protocol repositories (MEDLINE, Cochrane Database of Systematic Reviews, and JBI Evidence Synthesis) did not identify recent or ongoing systematic or scoping reviews with this specific population focus and concept, ensuring the originality of the review.

Therefore, this scoping review is justified by the need to map and synthesize the available evidence on vocal quality in individuals affected by COVID-19, with a special focus on mild and moderate cases, not subjected to orotracheal intubation. This scoping review aimed to map vocal changes in non-intubated adults affected by COVID-19, including mild to moderate cases, considering auditory-perceptual, acoustic, and self-reported changes in vocal quality, and situating the analysis in the clinical context of post-infection follow-up and studies published between 2019 and 2025, focusing on evidence that can support clinical practice, vocal rehabilitation strategies, and future research.

## METHODS

This scoping review followed the recommendations of the Preferred Reporting Items for Systematic Reviews and Meta-Analyses – extension for Scoping Reviews (PRISMA-ScR) and described by the Joanna Briggs Institute Reviewer’s Manual^(14-17)^. Its objectives, inclusion criteria, and methods were specified and documented in a protocol registered with the Open Science Framework (OSF)^(18)^.

### Eligibility criteria

The PCC (Population, Concept, and Context) strategy was used to develop the research question and eligibility criteria. The following key topics were adopted: the Population consisted of individuals aged 18 to 65 years with a diagnosis of COVID-19; the Concept was vocal quality in individuals with a diagnosis of COVID-19; and the context consisted of individuals with mild to moderate COVID-19 to exclude individuals who underwent endotracheal intubation during the acute phase of the disease. Considering the key topics of the PCC acronym in relation to the study objective, the following research question was formulated: "What are the characteristics of vocal quality in adults with a confirmed diagnosis of COVID-19?".

### Inclusion and exclusion criteria

Studies presenting original primary or secondary data on vocal quality in the population of interest were included, such as observational studies, case reports, and systematic reviews. Texts that did not present original data, such as theoretical essays, editorials, letters, and commentaries, were excluded because their focus is on argumentation and opinion, and they are not amenable to data extraction for evidence-based synthesis. Experience reports were retained as long as they systematically described cases or case series with vocal assessment. Studies that included participants who underwent endotracheal intubation during hospitalization for COVID-19, or who presented comorbidities such as smoking, previous history of voice disorders or laryngeal alterations, any form of respiratory, dermatological, rheumatic, or neurological disease with laryngeal involvement, history of head and neck cancer, or cervical radiotherapy were also excluded.

### Research sources and search strategy

The electronic search was conducted in June 2024 in six citation and abstract databases: Latin American and Caribbean Health Sciences Literature (LILACS), Medical Literature Analysis and Retrieval System Online (MEDLINE), EMBASE, Web of Science, Scopus, and Cochrane. A grey literature search was also conducted in January 2025 on Google Scholar, MedRxiv, and ProQuest. Citations of the selected articles were also searched. The search strategies were developed using indexed and free terms related to PCC. Combinations of descriptors related to COVID-19 (e.g., "COVID-19", "SARS-CoV-2") and vocal quality (e.g., "Voice", "Dysphonia", "Voice Quality") were used with Boolean operators (AND, OR) and truncations. The search was restricted to English due to the greater coverage of international databases for the investigated topic. The complete strategy per database is detailed in Appendix A.

### Selection

The studies were selected in four stages: (1) calibration (2) screening of titles and abstracts; (3) exclusion of duplicates; (4) full reading of eligible articles.

Studies were initially identified in the PubMed/MEDLINE and LILACS databases to calibrate the selection. Duplicate records were removed using automatic tools from the Rayyan portal. Calibration was performed by two reviewers independently. The calibration stage used a pilot sample of 82 articles, evaluated according to the inclusion and exclusion criteria. Cohen's Kappa coefficient was calculated to measure interrater agreement, establishing a minimum 0.7 coefficient as a criterion to advance to the main screening. After the calibration stage, the studies were identified in the LILACS, MEDLINE, EMBASE, Web of Science, Scopus, and COCHRANE databases. A grey literature search was also conducted in January 2025 on Google Scholar, MedRxiv, and ProQuest. Duplicate records were removed using automated tools from the Rayyan portal. The selection was carried out independently by the two reviewers. In the initial screening, inclusion criteria were applied by reading the title and abstract, and were manually screened by both reviewers. The second stage of selection consisted of reading the full articles and applying the exclusion criteria. A third researcher arbitrated any disagreements.

### Data extraction and storage

Data were extracted in a standardized manner by two independent reviewers using spreadsheets (Excel). The variables collected included author, year and country of publication, methodological design, study objective, sample size, sex, age group and race/ethnicity of participants, presence of previous comorbidities, COVID-19 diagnostic method, clinical classification of the disease (mild or moderate), vaccination status, signs and symptoms related to COVID-19, protocols and instruments used for vocal assessment, acoustic parameters, auditory-perceptual results, self-reported vocal symptoms, follow-up time, and main conclusions reported by the authors.

The synthesis was performed through descriptive and quantitative analysis, with calculation of absolute and relative frequencies of the extracted variables. The results were organized into tables and graphically represented in word clouds, distribution graphs, and geographic maps, when applicable. This approach sought to provide a comprehensive view of the available evidence, highlighting trends and gaps in the literature.

## RESULTS

The database search strategy retrieved 35,497 references, totaling 442 after removing duplicates using automated tools from the Rayyan portal and manually extracting ineligible references. The criteria excluded references based on document type, age range incompatible with the defined population (18-65 years), absence of mention of confirmed COVID-19, publication year incompatible with the defined population (2019-2025), and publication language (English, Portuguese, and Spanish). During the title and abstract reading phase, 109 publications were eligible for full-text reading. After reading, 90 studies were excluded, resulting in 19 publications included for the synthesis (Appendix B).

### Characteristics of the studies

**Figure 1:**
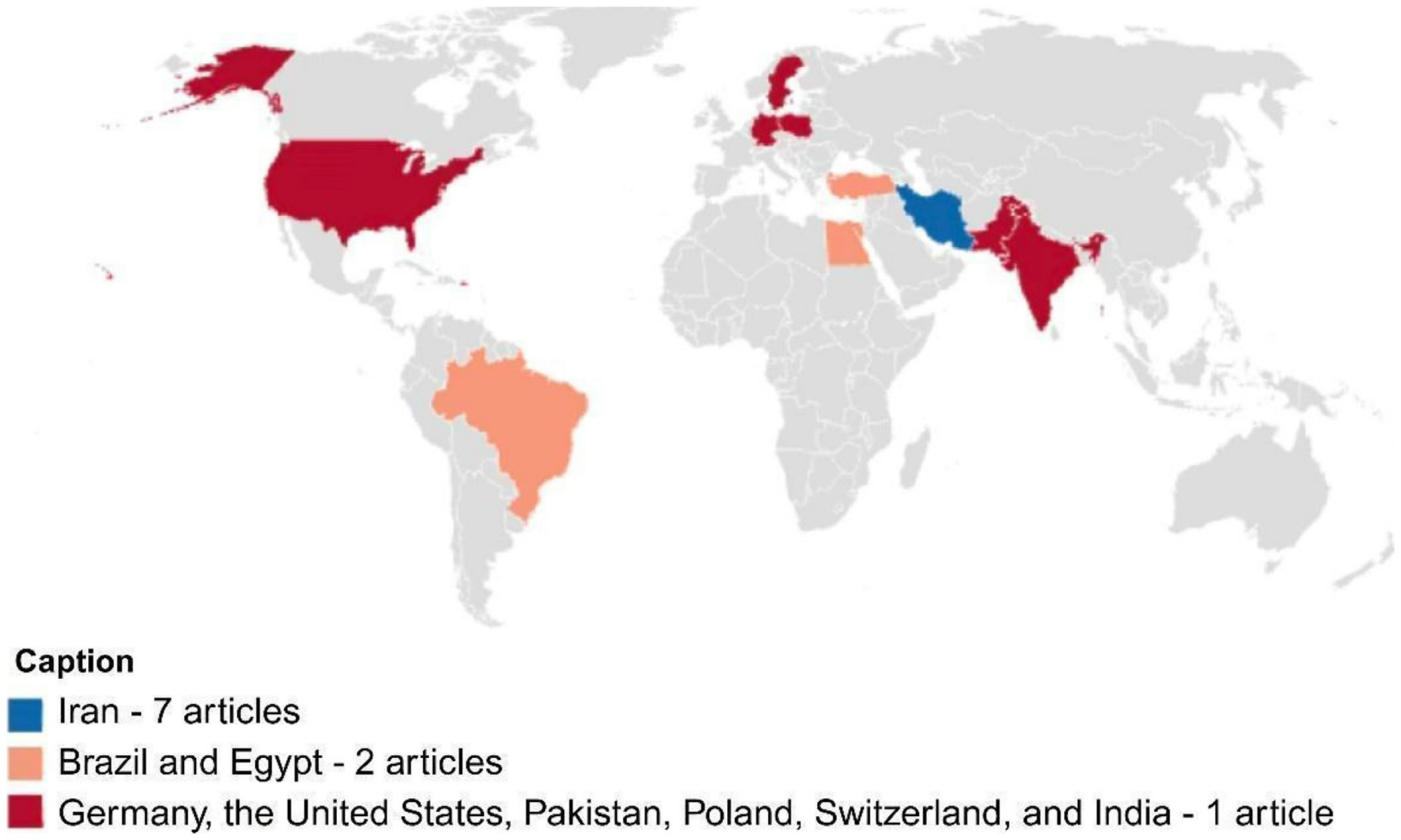
Distribution of articles included in the scope review by nationality

The review included 19 studies, published between 2020 and 2025, peaking in 2021 (n = 6) and 2022 (n = 5). Most studies were conducted in Iran (n = 7), followed by Turkey (n = 2), Egypt (n = 2), and Brazil (n = 2) (Figure 1). Other countries included Germany, the United States, Pakistan, Poland, Switzerland, and India (Table 1).

**Caption:** World map highlighting the nationality of the articles included in the scoping review. Each country is represented by a distinct color, according to the frequency of publications: **Blue (Iran):** 7 articles. **Dark orange (Brazil and Egypt):** 2 articles each. **Red (Germany, the United States, Pakistan, Poland, Switzerland, and India):** 1 article each

**Table 1:** Study characteristics - part I

**Author(s)**	Year	Country
Asiaee et al.^(19)^	2020	Iran
Helding et al.^(9)^	2020	USA
Eltelety and Nassar^(20)^	2020	Egypt
Korkmaz and Güven**^(^** ^21)^	2021	Turkey
Saki et al.^(22)^	2021	Iran
Bartl-Pokorny et al.^(23)^	2021	Germany
Tohidast et al.^(24)^	2021	Iran
Dassie-Leite et al.^(25)^	2021	Brazil
Azzam et al.^(26)^	2021	Egypt
Gölaç et al.^(12)^	2022	Turkey
Aghadoost et al.^(27)^	2022	Iran
Aghaz et al.^(11)^	2022	Iran
Shanem et al.^(28)^	2022	Pakistan
Khoddami et al.^(13)^	2023	Iran
Saki et al.^(29)^	2023	Iran
Kuć and Michta^(30)^	2023	Poland
Bueno et al.^(10)^	2024	Brazil
Rahul et al.^(31)^	2024	India
Berti et al.^(32)^	2025	Switzerland

Observational studies predominated: cross-sectional, analytical, and case control, with considerable methodological variations. Two case reports and one systematic review with meta-analysis were also included (Table 2).

**Table 2:** Study characteristics - part II

**Article**	**Study type**	**Study objective**	**Sample size**	**Number of men**	**Number of women**	**Age range**
Gölaç et al.^(12)^	observational, case-control	To compare the vocal outcomes of recovering patients with a control group.	80 (40 cases and 40 controls)	40	40	21 to 53 years
Korkmaz and Güven**^(^** ^21)^	Case report	To present a case of sudden unilateral vocal cord paralysis believed to be associated with COVID-19 infection.	1	0	1	55 years
Saki et al.^(22)^	Observational, case-control study	To conduct an auditory-perceptual evaluation of the vocal characteristics of patients with varying degrees of COVID-19 severity.	102	94	38	Patient group with a mean age of 58.1 ± 9.1 years. Healthy group with a mean age of 46.7 ± 9.3 years.
Bartl-Pokorny et al.^(23)^	case control	To provide a deeper understanding of the voice characteristics of patients with COVID-19.	22	14	8	With COVID: mean age +/- 60 years +/- 20 years standard deviation; age range +/- 19-79 years; Without COVID: mean age +/- 55 years +/- 20 years standard deviation; age range +/- 24-85 years
Khoddami et al.^(13)^	observational and cross-sectional, case-control	To determine general health and voice-related QoL and investigate the correlation between them in Iranian patients with COVID-19 and healthy individuals.	68 (34 controls and 34 cases)	20	14	18 to 50 years
Saki et al.^(29)^	cross-sectional, case-control	To compare signs and symptoms of vocal fatigue between patients with COVID-19 and people with normal voices.	60 (30 cases and 30 controls)	18 cases and 14 controls	12 cases and 16 controls	Mean age: 35.69 ± 6.92 years (controls) Mean age: 39.69 ± 10.42 years (cases)
Aghadoost et al.^(27)^	cross-sectional, case-control	To investigate the correlation between subjective analysis and objective analysis of voice in patients with COVID-19 infection.	80 (40 cases and 40 controls)	27 men per group	13 women per group	Case group: (mean age 41.2 ± 5.41 years). Control group: mean age 44.50 ± 3.50 years.
Tohidast et al.^(24)^	Case control	To investigate voice quality and symptoms of vocal tract discomfort in patients with COVID-19.	88 patients (44 cases and 44 controls)	24 per group	20 per group	The mean age of patients with COVID-19 and healthy individuals was 49.61 mean and 16.48 standard deviation and 48.52 mean and 13.8 standard deviation, respectively.
Dassie-Leite et al.^(25)^	Observational, analytical, cross-sectional, and hybrid	Compare the occurrence of vocal signs and symptoms before, during, and after COVID-19 and analyze possible risk factors for the persistence of these symptoms after resolution of the disease.	45	21	24	Mean age of 44 years and 10 months
Asiaee et al.^(19)^	Observational, case-control	To compare patients with COVID-19 with healthy individuals to assess the effect of this disease on acoustic parameters.	64 cases and 70 controls (134)	38 cases and 33 controls	26 cases and 37 controls	Mean age of 52.3 years in the case group and 42.35 years in the control group.
Helding et al.^(9)^	Review/Discussion Article	To discuss the long-term effects of COVID-19 and its impact on the voice, with a focus on singers.	-	-	-	-
Aghaz et al.^(11)^	systematic review and meta-analysis	To estimate the prevalence of dysphonia in patients with COVID-19.	1410	28.20%	32.80%	42.71 years
Azzam et al.^(26)^	Clinical study	To detect the occurrence of vocal symptoms in COVID-19 patients in Egypt and to investigate the associated videolaryngoscopy findings.	106	28	78	18 to 66 years
Bueno et al.^(10)^	Observational study	To identify vocal impairments and changes in voice-related quality of life in patients with pulmonary impairment associated with COVID-19.	35	22	13	18 to 65 years
Shanem et al.^(28)^	Retrospective cross-sectional study	To investigate the impact of COVID-19 on vocal cord function and voice production.	379	168	211	20 to 50 years
Kuć and Michta^(30)^	Observational study	To diagnose the types of speech disorders that occur in COVID-19 survivors.	30	15	15	30 to 60 years
Berti et al.^(32)^	Comparative study	To compare the voice and speech characteristics between post-COVID-19 individuals and control individuals.	134 (70 controls and 64 cases)	30 controls and 25 cases	40 controls and 39 cases	40 to 60 years
Eltelety and Nassar^(20)^	case study	To report a patient with COVID-19 presenting primarily with hoarseness and anosmia.	1	1	0	27
Rahul et al.^(31)^	Case control	To explore the perceptual changes, self-reported outcomes, and acoustic changes in the voice of individuals infected with COVID-19 immediately after the isolation period and compare them with the control group.	25	0	25	18 to 40 years

The studies aimed primarily to investigate the effects of COVID-19 on vocal quality through different assessments, including acoustic analysis^(12,19,20,23,27)^, auditory-perceptual evaluation^(22,24)^, self-perception protocols^(13,25,29)^, and instrumental examinations^(13,31)^. Several studies^(10,12,27,31)^ compared individuals with COVID-19 to healthy control groups, seeking to identify vocal changes, persistent symptoms, vocal fatigue, and impact on voice-related quality of life. Correlations between subjective and objective voice measures were also explored, as well as reports of specific clinical cases^(20,21)^, such as sudden vocal paralysis and mild manifestations such as hoarseness. Some studies^(9,10)^ highlighted specific populations, including singers or patients with pulmonary impairment, broadening the understanding of possible vocal sequelae associated with infection (Table 2).

Sample sizes ranged from one (case report) to 1,410 participants (meta-analysis), with ages ranging from 18 to 66 years, the majority being male (Table 2).

Some studies used only the reverse transcription polymerase chain reaction (RT-PCR) test performed in the laboratory^(10,12,21,22,25,27,29,30)^. Other studies performed the RT-PCR test associated with computed tomography (CT)^(13,19,24,28,33)^ or Rapid Antigen Test (RT-Ag) or Self-Antigen Test (SA-Ag)^(20)^.

### Clinical context found in the studies

The signs and symptoms of COVID-19 reported in the studies show a variety of clinical manifestations, with respiratory symptoms predominating (Table 3). Dry cough, dyspnea, and respiratory discomfort were the most frequently described, appearing in isolation or in association with mild to moderate pneumonia^(10,12,19,27)^. Fever and fatigue were also widely mentioned, with fatigue being a recurring complaint both in the acute phase and among patients with persistent symptoms^(25,29)^. Neurosensory symptoms, such as anosmia and dysgeusia, were present in several reports, in addition to headache, myalgia, and joint pain^(13,20,24)^. In some studies, the presence of vocal changes such as hoarseness, dysphonia, and vocal fatigue stood out, appearing both as an isolated symptom and in association with respiratory symptoms, even being the main indicator of laryngeal complications, such as vocal fold paralysis^(21,31)^. Other vocal symptoms included dry throat, throat clearing, and a sensation of thick mucus^(10,24)^.

**Table 3:** Clinical context found in the studies

**Article**	**COVID-19 classification**	**different vaccination statuses**	**COVID-19 signs and symptoms**	**Vocal signs and symptoms**
Gölaç et al.^(12)^	The article does not classify COVID-19.	Not reported	cough, shortness of breath, tiredness, difficulty speaking, and pain	Cough, shortness of breath, fatigue, difficulty speaking, and pain.
Korkmaz and Güven**^(^** ^21)^	The article does not classify COVID-19.	Vaccinated against COVID-19 with two doses of the vaccine before being diagnosed with the infection.	Cough and shortness of breath, fever, fatigue, progressive hoarseness.	Hoarseness (dysphonia), difficulty speaking, vocal fatigue
Saki et al.^(22)^	Mild, Moderate, Severe	Not reported	Not reported	Dysphonia, hoarseness, and difficulty controlling vocal production.
Bartl-Pokorny et al.^(23)^	The COVID participants presented with mild to moderate respiratory symptoms.	Not reported	Mild to moderate respiratory symptoms.	Not reported
Khoddami et al.^(13)^	mild to moderate	Not reported	Absence of pneumonia, mild complications, fever, signs of pneumonia, and infection.	Not specified
Saki et al.^(29)^	Not classified	Not specified	Cough, pharyngitis, sore throat, and difficulty breathing.	Vocal fatigue, hoarse voice, breathy voice, vocal strain, and voice breaks.
Aghadoost et al.^(27)^	Mild to moderate COVID-19	Not reported	Pneumonia, fever, and other respiratory symptoms, but no respiratory failure requiring mechanical ventilation.	Dysphonia (voice alterations, such as hoarseness), reduction in vocal quality, with increased roughness, breathiness, tension and vocal effort, reduction in maximum phonation time
Tohidast et al.^(24)^	Mild, moderate and severe (ICU)	Not reported	Not reported	Vocal discomfort (burning, pain, dryness, irritability and lump in the throat, tightness)
Dassie-Leite et al.^(25)^	Mild, acute, severe, critical	Nor reported	cough, fever, dyspnea, musculoskeletal symptoms (myalgia, joint pain, fatigue), gastrointestinal symptoms, anosmia and dysgeusia.	laryngeal sensitivity and vocal changes, dysphonia,
Asiaee et al.^(19)^	Not reported	Not reported	Not reported	Not specified
Helding et al.^(9)^	Not applicable	Not applicable	respiratory, neurological and post-infectious symptoms	Review of evidence
Aghaz et al.^(11)^	Not reported	Not reported	Specific signs and symptoms are not detailed.	Dysphonia
Azzam et al.^(26)^	mild to moderate	Not reported	dysphonia, phonesthenia, nasal obstruction, loss of smell, taste dysfunction, sore throat, sticky mucus, and dysphagia	Dysphonia and phonesthenia
Bueno et al.^(10)^	Not reported	Not reported	dyspnea and cough	dysphonia, vocal fatigue and laryngospasm
Shanem et al.^(28)^	Not reported	Not reported	Not reported	Weak and trembling voice, unclear voice and vocal changes
Kuć and Michta^(30)^	Not reported	Not reported	Not reported	hoarseness, cough, pain when swallowing, and a feeling of a foreign body in the throat
Berti et al.^(32)^	mild to moderate	Not reported	Runny nose, sore throat, cough, fever, headache, loss of taste/smell, fatigue, muscle aches and/or diarrhea.	Not reported
Eltelety and Nassar^(20)^	Not reported	Not reported	Hoarseness and anosmia	Hoarseness
Rahul et al.^(31)^	Not reported	Not reported	Cough, dry throat, and phlegm.	difficulty increasing volume and other vocal problems

The vocal signs and symptoms associated with COVID-19, as reported in the studies, encompass a variety of perceptual, acoustic, and self-perceptual changes (Table 3). Dysphonia, often described as hoarseness, was the most recurrent vocal symptom and, in some cases, the main complaint that motivated the clinical investigation, even being the first symptom perceived by some participants^(11,12,21,23)^. In addition to hoarseness, other reported symptoms included difficulty speaking clearly, weak, trembling, strained, breathy, and raspy voice, vocal tension, changes in vocal intensity, and changes in the overall quality of the voice^(24,27,28,31)^. Symptoms such as vocal fatigue, pain, burning, dryness, a foreign body sensation, and tightness in the throat were also highlighted, evidencing persistent laryngeal discomfort^(10,24,29)^.

**Figure 2:**
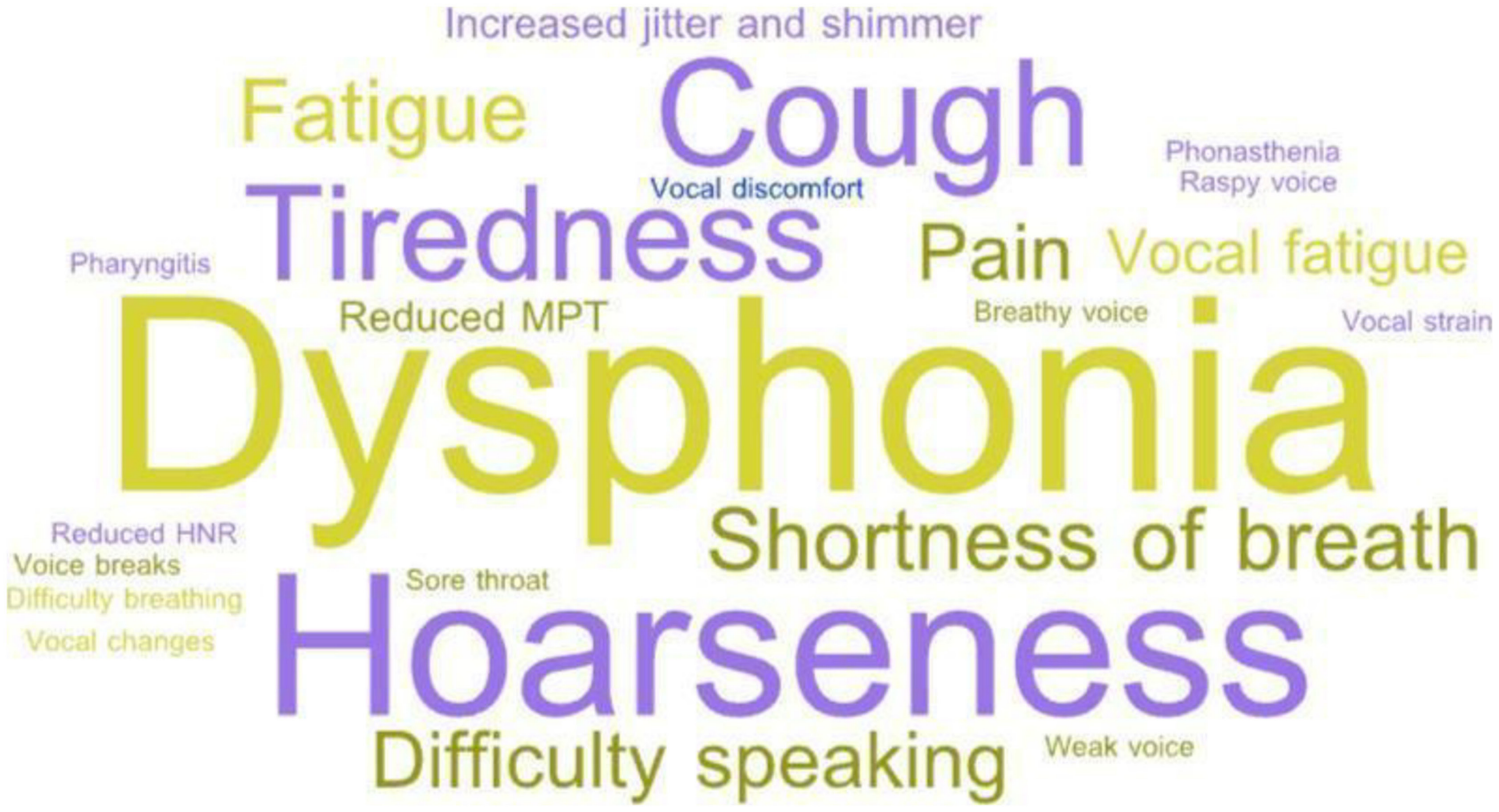
Vocal signs and symptoms associated with COVID-19

Figure 2 shows a word cloud highlighting the most frequent vocal signs and symptoms associated with COVID-19, emphasizing terms such as hoarseness, vocal fatigue, and voice changes.

**Caption:** The word cloud shows the main vocal symptoms reported in the analyzed studies, such as dysphonia, hoarseness, vocal fatigue, reduced maximum phonation time (MPT), and vocal fold paralysis

### Vocal assessments found in studies

**Figure 3:**
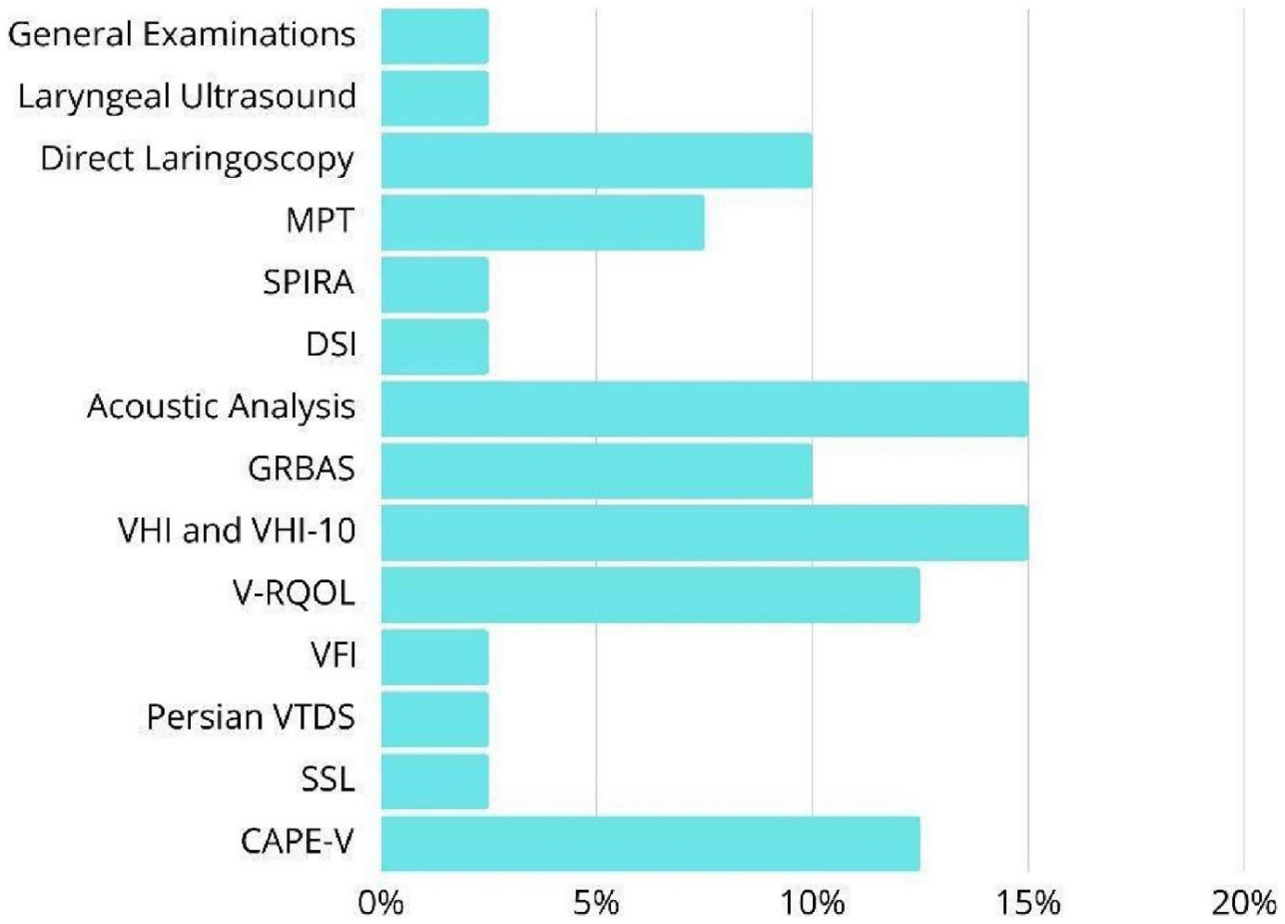
Frequency of vocal assessment protocols used

The included studies used a variety of methods and instruments to assess the vocal function of individuals affected by COVID-19, predominantly acoustic analyses, auditory-perceptual evaluation, and self-perception (Figure 3). Measures such as maximum phonation time (MPT), jitter, shimmer, fundamental frequency (F0), minimum intensity, and harmonic-to-noise ratio (HNR) stood out in acoustic analysis^(19,23,27,31)^. Some studies calculated the dysphonia severity index (DSI) as a composite measure of dysphonia severity^(12,27)^ (Figure 3). Auditory-perceptual evaluation was mainly conducted using the CAPE-V and GRBAS protocols, including tasks of sustained vowel emission, sentence reading, and spontaneous speech^(24,29)^. The CAPE-V was applied in different languages ​​and contexts, focusing on parameters such as roughness, breathiness, strain, and overall severity^(12,27)^ (Figure 3).

**Caption:** The figure illustrates the most used questionnaires and protocols for assessing voice-related quality of life: VHI-10 (Voice Handicap Index-10), V-RQOL (Voice-Related Quality of Life), VFI (Vocal Fatigue Index), and VTD (Vocal Tract Discomfort Scale)

As for self-reported instruments, the most used questionnaires were the Voice Handicap Index (VHI-10)^(10,12,13)^, which assesses the impact of dysphonia on daily life, and the Voice-Related Quality of Life (V-RQOL)^(13,25)^, which measures quality of life regarding the voice. The Vocal Fatigue Index (VFI)^(24,29)^ and the Vocal Tract Discomfort Scale (VTD)^(24,25)^ were also used, in addition to the SSL (list of vocal signs and symptoms)^(25)^ at different times during infection (before, during, and after COVID) (Figure 3). Additionally, some studies^(20,21,26)^ included examinations such as laryngoscopy and laryngeal ultrasound to investigate the anatomy and mobility of the vocal folds, especially in cases of laryngeal paralysis. Although not all studies specified the protocols used, there was a general tendency to combine different methods to provide a more comprehensive and sensitive vocal assessment of the effects of COVID-19.

Acoustic analysis was widely used in the reviewed studies (Table 4) to objectively assess changes in vocal quality in individuals who recovered from COVID-19. Several parameters were measured using software such as Praat^(12,19,32)^ and VoiceSauce^(19)^, using both sustained phonation and spontaneous speech.

**Table 4:** Vocal assessments found in studies

**Article**	**Vocal assessment**	**Acoustic analysis**
Gölaç et al.^(12)^	Acoustic Analysis	Acoustic Parameters Evaluated:
	Aerodynamic Measurement: MPT	Average Fundamental Frequency (F0)
	Self-Perception Questionnaires: Voice Handicap Index-10 (VHI-10), Voice-Related Quality of Life (V-RQOL)	Local Jitter
	Auditory-Perceptual Evaluation: CAPE-V	Local Shimmer
		Harmonic-to-Noise Ratio (HNR)
		Cepstral Peak Prominence (CPP).
		Methods and Tools Used:
		Software: Acoustic analyses were performed using Praat software (version 6.0.17).
		Voice Samples: collected through sustained phonation of the vowel /a/ and linked speech (third sentence of the CAPE-V protocol in Turkish).
		Analyzed Segment: For perturbation measures (jitter, shimmer, and HNR), the middle segment of the vowel /a/ in the most stable part of phonation was used. For the CPP, the third sentence of the CAPE-V protocol was analyzed.
Korkmaz and Güven**^(^** ^21)^	Acoustic analysis	Fundamental Frequency (F0): The fundamental frequency, or pitch, was measured to verify the variation in voice tone, since unilateral paralysis can affect the symmetry of vocal cord movement and, consequently, the frequency of vocal emission.
	Voice Handicap Index (VHI) assessment	Jitter: refers to the variation in the fundamental frequency of the voice and can be used to assess the stability of vocal tone. Paralysis can cause fluctuations in tone, reflecting vocal irregularity.
	Laryngoscopy: Direct laryngoscopy was performed to examine the vocal cords and identify unilateral paralysis.	Shimmer: measures the variation in sound amplitude, indicating the regularity of vocal intensity. Vocal cord paralysis can affect the consistency of vocal production, altering the shimmer.
	Aerodynamic measurement: MPT, using the s/z ratio.	Vocal Quality Index: Parameters such as sound regularity (measured by jitter and shimmer) are frequently used to assess vocal quality. In the case of vocal paralysis, these parameters may be altered, reflecting compromised vocal quality.
Saki et al.^(22)^	Auditory-Perceptual Evaluation: GRBAS	Acoustic analysis was performed to evaluate parameters such as fundamental frequency (F0), intensity, and disturbances in voice frequency and amplitude, providing a more objective assessment of the alterations.
Bartl-Pokorny et al.^(23)^	This study analyzed recordings of sustained vowels (/i:/, /e:/, /o:/, /u:/, and /a:/) produced by German-speaking participants. The research focused on 88 acoustic characteristics extracted from these recordings, without specifying traditional vocal assessment protocols such as maximum phonation duration or vocal intensity. Statistical analysis was performed using the Mann-Whitney U test and effect size calculations to identify significant differences between the participant groups.	The acoustic analysis included the following parameters:
		Fundamental frequency (F0): The average frequency of the voice, which can reflect changes in vocal control and vocal cord tension.
		Harmonic-to-noise ratio (HNR): A measure that indicates the clarity of the voice. The lower the HNR ratio, the greater the presence of noise in the voice.
		Mel's cepstral coefficients (MFCC): Spectral characteristics that help describe the acoustic content of the voice in a way that simulates human auditory perception.
		Spectral slope: Reflects how energy is distributed across the frequencies of the vocal sound.
		Vowel duration and number of sound segments per second: Indicators of the rhythm and fluency of vocal production.
Khoddami et al.^(13)^	Voice Handicap Index (VHI): This index is a widely used tool to assess the impact of vocal dysfunction on individuals' quality of life. It helps measure the degree of discomfort and limitation that vocal problems cause in patients' daily lives.	It does not mention whether it conducted acoustic analyses of the participants' voices.
	Health-related quality of life assessment: Although the study does not provide specific details on the exact protocol used to measure quality of life, it is mentioned that quality of life assessment was an important part of the analysis, comparing patients recovered from COVID-19 with healthy subjects.	
Saki et al.^(29)^	Acoustic Evaluation of the Voice	Evaluated Parameters: Jitter (%), Shimmer (%), MPT (seconds), HNR (dB).
	Auditory-perceptual evaluation: CAPE-V	Results:
	Self-perception questionnaire: Vocal Fatigue Index	Patients with COVID-19 presented higher Jitter, Shimmer, and lower HNR and MPT compared to the control group.
		Prolonged voice use (after reading the text) exacerbated vocal irregularities in patients with COVID-19.
		Correlations:
		The acoustic parameters (Jitter, Shimmer, HNR) correlated significantly with vocal fatigue (voice tiredness and physical discomfort).
Aghadoost et al.^(27)^	Auditory-perceptual evaluation: CAPE-V	Frequency Disturbance (Jitter): Higher Fundamental Frequency (F0 high)
	Self-perception questionnaire: Dysphonia Severity Index (DSI)	
Tohidast et al.^(24)^	Auditory-perceptual evaluation: GRBAS	Not performed
	Self-perception questionnaire: VTD (vocal tract discomfort)	
Dassie-Leite et al.^(25)^	Vocal Signs and Symptoms list (SSL), which investigates the presence or absence of 14 vocal signs or symptoms. A translation of the instrument into Brazilian Portuguese was used. Each symptom was addressed in relation to three distinct moments: before, during, and after COVID-19.	Not performed
Asiaee et al.^(19)^	Acoustic analysis	A Praat script was used to extract the local values ​​of jitter, shimmer, MPT, and the number of voice breaks. Measurements were performed using the default settings in Praat. Fundamental frequency, CPP, HNRs, and (H1-H2) were automatically extracted using VoiceSauce, a freeware for voice analysis.
		The F0 measurement was performed using the standard VoiceSauce algorithm, namely STRAIGHT. In VoiceSauce, CPP is calculated using the algorithm proposed by Hillenbrand et al. HNR values ​​are measured using a variable window of five pitch periods by Krom's algorithm. HNR05, HNR15, HNR25, and HNR35 measure HNR from 0-500 Hz, 0-1500 Hz, 0-2500 Hz, and 0-3500 Hz, respectively. This study used HNR35 (hereinafter HNR).
		Finally, H1*-H2* was used for evaluation (H1-H2), which is H1-H2 corrected for the formant effect based on the algorithm proposed by Iseli et al. and used in VoiceSauce.
Helding et al.^(9)^	Review of studies using acoustic and perceptual methods	Not applicable
Aghaz et al.^(11)^	The contexts do not provide specific vocal assessment protocols used in the studies.	The contexts do not provide details about any acoustic analysis performed..
Azzam et al.^(26)^	Includes auditory-perceptual evaluation of voice and videolaryngoscopy examination.	Not mentioned in the contexts provided.
Bueno et al.^(10)^	Voice Handicap Index 10 (VHI-10) and the Voice-Related Quality of Life (VQOL) and MPT protocol	Not specified in the contexts provided.
Shanem et al.^(28)^	The Voice Handicap Index (VHI) was used to assess voice concerns.	Not performed
Kuć and Michta^(30)^	It included recordings of spontaneous speech, repetition of words and phrases, a monologue, and automated word sequences; perceptual assessment was conducted using the GRBAS scale.	Not specified in the contexts provided.
Berti et al.^(32)^	The recordings were made using SPIRA software based on three verbal tasks: sustained production of the vowel /a/, reading a sentence, and producing a rhyme.	Conducted using PRAAT software, focusing on various acoustic parameters.
Eltelety and Nassar^(20)^	A follow-up transcervical laryngeal ultrasound was performed to assess vocal fold mobility.	Not specified
Rahul et al.^(31)^	Perceptual assessment, self-reported results using the Voice Handicap Index (VHI-10), and acoustic analysis.	The study revealed significant differences in acoustic parameters between the experimental and control groups.

The review points out that COVID-19 infection can negatively impact vocal quality, even after the acute phase of the disease^(12,25)^. The studies often found auditory-perceptual changes, such as roughness, strain, and overall deviation of vocal quality, in addition to impairments in self-perception measures, with high scores on instruments such as VHI-10 and V-RQOL^(13,24,25,27)^. Although many studies have not found statistically significant differences in acoustic parameters such as F0, jitter, and shimmer, an MPT reduction was common^(19,23)^. The presence of symptoms such as cough, dyspnea, and fatigue during COVID-19 correlated with greater perceived vocal impact^(12,29)^. Cases of vocal fold paralysis and neurological changes have also been reported, suggesting possible central and peripheral mechanisms involved in post-COVID voice disorders^(20,21)^.

Data for the synthesis were organized by study characteristics, type of vocal assessment, and clinical context, considering the methodological variability of the studies and highlighting consistent and discrepant evidence.

## DISCUSSION

The 19 publications included in this study show that COVID-19 is associated with significant vocal changes, even in mild to moderate cases that did not undergo endotracheal intubation. The findings corroborate previous studies that highlight the multifactorial impact of SARS-CoV-2 on the respiratory, phonatory, and neurological systems, resulting in voice disorders^(5,6,29)^.

Seven studies (36.8%) included in the review were conducted in the Islamic Republic of Iran. According to the World Health Organization (WHO), as of February 28, 2022, the country had recorded more than 6.6 million confirmed cases of COVID-19 and approximately 140,000 deaths^(33)^. During the pandemic, Iran faced significant limitations in its capacity to perform genomic sequencing, which hampered epidemiological surveillance and the tracking of new SARS-CoV-2 variants, negatively impacting the effectiveness of the national response to the health crisis. In response to these limitations, WHO and its international partners provided technical and financial support to the country, promoting the acquisition of advanced technology equipment, specific software, and technical training. In addition, comprehensive risk communication campaigns and community engagement strategies were implemented to foster a community-centered response, encouraging the demand for testing, treatment, and vaccination^(33)^.

The included studies totaled 2,730 participants, predominantly male. Despite the relevance of the race/skin color variable for understanding health inequalities, none of the included studies performed analysis by race or ethnicity. This lack of data limits the analysis of possible racial disparities in the vocal impact of COVID-19. The literature has shown that racially marginalized groups were more affected by the pandemic in terms of infection, severity, and access to healthcare, partly due to structural factors such as socioeconomic inequality, working conditions, and less access to post-COVID rehabilitation^(34)^. Thus, the systematic exclusion of race as a variable in studies on vocal quality prevents the identification of potentially more vulnerable populations.

Dysphonia was the most described symptom, with reported frequency ranging from 25% to 79%, depending on the sample and criteria adopted in the studies^(8,11,12)^. The meta-analysis conducted by Aghaz et al.^(11)^ indicated a combined prevalence of 25.1% during the acute phase of the disease, with persistence of vocal symptoms in 17.1% after recovery. Other studies reported even higher percentages, suggesting that the assessment methods and clinical characteristics of the cohorts substantially influence the findings^(8)^. A higher prevalence trend of dysphonia was also observed among female participants, as indicated in the meta-analysis by Aghaz et al.^(11)^ and in observational studies included in this review^(10,13,25)^. These data suggest that, in addition to the frequency of symptoms, the functional impact of dysphonia may be more pronounced in females. This difference can be attributed to anatomical and hormonal factors — such as thinner vocal folds and faster glottal vibration — that make the female larynx potentially more vulnerable to changes. In addition, behavioral and social aspects may lead women to perceive and report vocal symptoms more frequently, reflecting greater sensitivity to changes in voice quality.

Acoustic analysis revealed an increase in jitter and shimmer and a decrease in MPT, indicating vocal instability and possible respiratory impairment^(19,23)^. Asiaee et al.^(19)^ found a statistically significant difference in acoustic parameters between individuals recovered from COVID-19 and participants in the control group, composed of healthy individuals with no history of the disease. In addition, the MPT, a measure of respiratory efficiency and glottal competence, was systematically reduced in the groups affected by the infection^(10,12,25)^. Gölaç et al.^(12)^ found an average MPT of 11.9 seconds in post-COVID-19 patients, in contrast to 16.4 seconds in healthy controls. Similarly, Bueno et al.^(10)^ observed an MPT of 8.9 seconds in patients with severe pneumonia and 10.7 seconds in those with moderate pneumonia, compared to 16.2 seconds in healthy controls^(10)^. These findings corroborate the hypothesis that COVID-19 can compromise the larynx and the respiratory support necessary for voice production. In addition, the reduction in HNR suggests a greater presence of noise in the voice, associated with laryngeal inflammation or muscle weakness^(20)^. Participants reported persistent vocal fatigue, difficulty projecting their voice, and a feeling of effort when speaking^(22,25)^.

Instruments such as the VHI-10 and V-RQOL confirmed that these changes negatively impact quality of life, especially in occupational voice users^(12,13)^. This shows that, even in cases where dysphonia is mild from a clinical point of view, it can have a significant functional impact, affecting everyday communication and quality of life, especially in individuals who depend on their voice to work.

Vocal changes can be attributed to multiple factors with respiratory impairment: reduced MPT and weak voice are associated with decreased lung capacity after COVID-19^(28)^; laryngeal inflammation: persistent cough and gastroesophageal reflux secondary to infection can cause edema and irritation of the vocal folds^(31)^; peripheral neuropathy: cases of vocal fold paralysis^(21)^ and changes in laryngeal innervation suggest that SARS-CoV-2 can affect cranial nerves; and generalized muscle fatigue: post-COVID-19 syndrome can lead to weakness of the respiratory and laryngeal muscles, affecting voice projection^(9)^.

Other frequently reported symptoms included dry throat, throat pain or burning, a sensation of thick mucus, and vocal fatigue^(10,24,29)^. In studies that used the Vocal Tract Discomfort Scale (VTDS), the sensation of dryness stood out with high scores and a large effect size, suggesting that alterations in laryngeal lubrication or inflammation are relevant components of the subjective experience of dysphonia^(28)^.

The results highlight the need for systematic vocal assessment in patients recovered from COVID-19, using protocols such as CAPE-V, GRBAS, and acoustic analyses^(24,27)^; early intervention, including speech therapy for respiratory and vocal rehabilitation, especially in cases of persistent dysphonia^(32)^; and multidisciplinary follow-up, involving otolaryngologists, speech-language-hearing pathologists, and pulmonologists, to manage integrated sequelae^(30)^.

The persistent vocal manifestations observed in some participants suggest a possible link to post-COVID syndrome, also called long COVID. WHO^(8)^ recognizes the presence of prolonged symptoms beyond 12 weeks after the onset of infection as a clinical criterion, and dysphonia may be among these long-lasting complaints, as observed in studies included in this review^(10,24,25,29)^.

The methodological heterogeneity among the studies, including vocal assessment protocols, instruments used, inclusion criteria, and follow-up periods, limits the generalizability of the results. Most studies presented short-term follow-up and restricted samples, making longitudinal analysis of vocal changes difficult.

The review did not formally assess the methodological quality of the included studies, which constitutes a limitation. In addition, potential biases in the search and selection of studies, as well as the exclusion of articles in languages ​​other than English, Portuguese, and Spanish, may have influenced the findings.

The results reinforce the importance of systematic vocal assessment in post-COVID-19 patients, using standardized protocols (CAPE-V, GRBAS, acoustic analyses) and self-report instruments. Early intervention, including speech therapy for respiratory and vocal rehabilitation, and multidisciplinary follow-up are recommended to mitigate functional sequelae.

Future studies should be longitudinal, multicenter, and with larger samples, including analyses by sex, race/ethnicity, and relevant clinical variables, to understand the evolution of vocal changes, pathophysiological mechanisms, and effective rehabilitation strategies. Furthermore, standardized protocols can reduce methodological heterogeneity and facilitate comparisons between different populations and clinical settings.

## CONCLUSION

This scoping review highlights that COVID-19 is associated with objective and subjective vocal changes, with functional and quality-of-life impact.

This study reinforces the importance of voice as a functional marker in COVID-19 and the need for a multidisciplinary approach to the management of these patients.

Further longitudinal studies are recommended to assess duration and rehabilitation in cases of post-COVID-19 vocal changes.

## Appendix A Search strategy

**Table:**

**Database**	**Date**	**Search strategy**	**Number of articles**
MEDLINE/PubMed	June 2024	((“COVID-19”[Mesh] OR "2019 Novel Coronavirus Disease”[tiab] OR "2019 Novel Coronavirus Infection”[tiab] OR "2019-nCoV Disease”[tiab] OR "2019-nCoV Infection”[tiab] OR “COVID19 Coronavirus Disease “[tiab] OR "Coronavirus Disease-19”[tiab] OR "SARS Coronavirus 2 Infection”[tiab] OR "SARS-CoV-2 Infection”[tiab] OR "Severe Acute Respiratory Syndrome Coronavirus 2 Infection”[tiab] OR "Covid 19"[tiab] OR "covid-19"[tiab] OR “covid19"[tiab] OR “coronavirus"[tiab] OR “SARS-COV-2”[tiab] OR "SARS-COV2" [tiab] OR "COVID disease”[tiab] OR “COVID sequelae”[tiab] OR ”COVID complications”[tiab] OR “COVID survivors”[tiab] OR “COVID infection” [tiab] OR “COVID infections”[tiab]) AND ("Voice Quality”[Mesh] OR “Voice”[Mesh] OR "Voice Qualities”[tiab] OR “Voices"[tiab] OR "Voice Disorders“[tiab] OR “Voice Disturbance “[tiab] OR “Voice Disturbances “[tiab] OR “Voice”[tiab] OR "Vocal quality”[tiab] OR "Voice quality”[tiab] OR "vocal health”[tiab] OR "voice health”[tiab] OR "vocal disorder”[tiab] OR "voice production”[tiab] OR "voice pathology”[tiab] OR "voice dysfunction"[tiab] OR "vocal dysfunction”[tiab]))	1,509
EMBASE	June 2024	('covid 19'/exp OR '2019 novel coronavirus disease':ti,ab OR '2019 novel coronavirus infection':ti,ab OR '2019-ncov disease':ti,ab OR '2019-ncov infection':ti,ab OR 'covid19 coronavirus disease':ti,ab OR 'coronavirus disease-19':ti,ab OR 'sars coronavirus 2 infection':ti,ab OR 'sars-cov-2 infection':ti,ab OR 'severe acute respiratory syndrome coronavirus 2 infection':ti,ab OR 'covid 19':ti,ab OR 'covid-19':ti,ab OR 'covid19':ti,ab OR 'coronavirus':ti,ab OR 'sars-cov-2':ti,ab OR 'sars-cov2':ti,ab OR 'covid disease':ti,ab OR 'covid sequelae':ti,ab OR 'covid complications':ti,ab OR 'covid survivors':ti,ab OR 'covid infection':ti,ab OR 'covid infections':ti,ab) AND ('voice quality'/exp OR 'voice'/exp OR 'voice qualities':ti,ab OR 'voices':ti,ab OR 'voice disorders':ti,ab OR 'voice disturbance':ti,ab OR 'voice disturbances':ti,ab OR 'voice':ti,ab OR 'vocal quality':ti,ab OR 'voice quality':ti,ab OR 'vocal health':ti,ab OR 'voice health':ti,ab OR 'vocal disorder':ti,ab OR 'voice production':ti,ab OR 'voice pathology':ti,ab OR 'voice dysfunction':ti,ab OR 'vocal dysfunction':ti,ab)	1,765
LILACS	June 2024	((mh:("COVID-19" OR "Voice Quality" OR "Voice")) OR (tw:("2019 Novel Coronavirus Disease" OR "2019 Novel Coronavirus Infection" OR "2019-nCoV Disease" OR "2019-nCoV Infection" OR "COVID19 Coronavirus Disease" OR "Coronavirus Disease-19" OR "SARS Coronavirus 2 Infection" OR "SARS-CoV-2 Infection" OR "Severe Acute Respiratory Syndrome Coronavirus 2 Infection" OR "Covid 19" OR "covid-19" OR "covid19" OR "coronavirus" OR "SARS-COV-2" OR "SARS-COV2" OR "COVID disease" OR "COVID sequelae" OR "COVID complications" OR "COVID survivors" OR "COVID infection" OR "COVID infections"))) AND ((mh:("Voice Quality" OR "Voice")) OR (tw:("Voice Qualities" OR "Voices" OR "Voice Disorders" OR "Voice Disturbance" OR "Voice Disturbances" OR "Voice" OR "Vocal quality" OR "Voice quality" OR "vocal health" OR "voice health" OR "vocal disorder" OR "voice production" OR "voice pathology" OR "voice dysfunction" OR "vocal dysfunction")))	279
SCOPUS	June 2024	( ( INDEXTERMS ( "COVID-19" ) OR INDEXTERMS ( "Voice Quality" ) OR INDEXTERMS ( "Voice" ) ) OR ( TITLE-ABS-KEY ( "2019 Novel Coronavirus Disease" OR "2019 Novel Coronavirus Infection" OR "2019-nCoV Disease" OR "2019-nCoV Infection" OR "COVID19 Coronavirus Disease" OR "Coronavirus Disease-19" OR "SARS Coronavirus 2 Infection" OR "SARS-CoV-2 Infection" OR "Severe Acute Respiratory Syndrome Coronavirus 2 Infection" OR "Covid 19" OR "covid-19" OR "covid19" OR "coronavirus" OR "SARS-COV-2" OR "SARS-COV2" OR "COVID disease" OR "COVID sequelae" OR "COVID complications" OR "COVID survivors" OR "COVID infection" OR "COVID infections" ) ) ) AND ( ( INDEXTERMS ( "Voice Quality" ) OR INDEXTERMS ( "Voice" ) ) OR ( TITLE-ABS-KEY ( "Voice Qualities" OR "Voices" OR "Voice Disorders" OR "Voice Disturbance" OR "Voice Disturbances" OR "Voice" OR "Vocal quality" OR "Voice quality" OR "vocal health" OR "voice health" OR "vocal disorder" OR "voice production" OR "voice pathology" OR "voice dysfunction" OR "vocal dysfunction" ) ) )	20,000
Web of science	June 2024	((TS=("2019 Novel Coronavirus Disease" OR "2019 Novel Coronavirus Infection" OR "2019-nCoV Disease" OR "2019-nCoV Infection" OR "COVID19 Coronavirus Disease" OR "Coronavirus Disease-19" OR "SARS Coronavirus 2 Infection" OR "SARS-CoV-2 Infection" OR "Severe Acute Respiratory Syndrome Coronavirus 2 Infection" OR "Covid 19" OR "covid-19" OR "covid19" OR "coronavirus" OR "SARS-COV-2" OR "SARS-COV2" OR "COVID disease" OR "COVID sequelae" OR "COVID complications" OR "COVID survivors" OR "COVID infection" OR "COVID infections")) AND (TS=("Voice Qualities" OR "Voices" OR "Voice Disorders" OR "Voice Disturbance" OR "Voice Disturbances" OR "Voice" OR "Vocal quality" OR "Voice quality" OR "vocal health" OR "voice health" OR "vocal disorder" OR "voice production" OR "voice pathology" OR "voice dysfunction" OR "vocal dysfunction")))	2,858
Cochrane	June 2024	#1 MeSH descriptor: COVID-19 #2 MeSH descriptor: Voice Quality #3 MeSH descriptor: Voice #4 :ti,ab,kw "2019 Novel Coronavirus Disease" OR "2019 Novel Coronavirus Infection" OR "2019-nCoV Disease" OR "2019-nCoV Infection" OR "COVID19 Coronavirus Disease" OR "Coronavirus Disease-19" OR "SARS Coronavirus 2 Infection" OR "SARS-CoV-2 Infection" OR "Severe Acute Respiratory Syndrome Coronavirus 2 Infection" OR "Covid 19" OR "covid-19" OR "covid19" OR "coronavirus" OR "SARS-COV-2" OR "SARS-COV2" OR "COVID disease" OR "COVID sequelae" OR "COVID complications" OR "COVID survivors" OR "COVID infection" OR "COVID infections" #5 :ti,ab,kw "Voice Qualities" OR "Voices" OR "Voice Disorders" OR "Voice Disturbance" OR "Voice Disturbances" OR "Voice" OR "Vocal quality" OR "Voice quality" OR "vocal health" OR "voice health" OR "vocal disorder" OR "voice production" OR "voice pathology" OR "voice dysfunction" OR "vocal dysfunction" #6 #1 OR #2 OR #3 OR #4 #7 #5 #8 #6 AND #7	474
Gray Literature	Date	Search strategy	Number of articles
ProQuest	January 2025	("COVID-19") AND ("Voice Quality")	3,103
MedRxiv	January 2025	("COVID-19") AND ("Voice Quality")	1,349
Google Scholar	January 2025	("COVID-19") AND ("Voice Quality”) 2019-2025	4,160

## Appendix B PRISMA 2020 Flowchart

Flowchart of the search and selection of relevant studies. Source: Search data.

*From:* Page et al.^(17)^.
