# ERRATUM: COVID-19-related voice disorders: a scoping review

**DOI:** 10.1590/2317-1782/e20250461en

**Published:** 2026-07-24

Due to an honest mistake by the authors, the article “COVID-19-related voice disorders: a scoping review” (DOI https://doi.org/10.1590/2317-1782/e20250243en), present in CoDAS 2026;38(3):e20250243, was published without identifying all authors.

**Where it was previously identified:**

Aline de Souza Silva, Rodrigo Dornelas, José Roberto Lapa e Silva.

**Now identify:**

Aline de Souza Silva, Isabella Marins Cassiano do Nascimento, Rodrigo Dornelas, José Roberto Lapa e Silva.

The authors apologize for the error.

Isabella Marins Cassiano do Nascimento

**ORCID:** https://orcid.org/0009-0000-7634-5310

**Affiliation:** Faculdade de Medicina, Universidade Federal do Rio de Janeiro – UFRJ - Rio de Janeiro (RJ), Brasil.

**Contribution:** conceptualization, data curation, research, methodology, writing, original draft, writing.

