# Supplementary material for: COVID-19-related voice disorders: a scoping review
Source: Codas. 2026 May 8;38(3):e20250243. doi: 10.1590/2317-1782/e20250243en (PMC13193193; doi:10.1590/2317-1782/e20250243en)
Supplement: Translation

# Translation: Disfunções vocais relacionadas à COVID-19: uma revisão de escopo

**DOI:** 10.1590/2317-1782/e20250243pt

**Authors:** Aline de Souza Silva, Rodrigo Dornelas, José Roberto Lapa e Silva

**Affiliations:** 1 Faculdade de Medicina, Universidade Federal do Rio de Janeiro – UFRJ - Rio de Janeiro (RJ), Brasil.

**Keywords:** COVID-19, Distúrbios da Voz, Fonoaudiologia, Infecções por Coronavirus, Qualidade da Voz, Revisão de Escopo

## Abstract

**Objetivo:**

Mapear as evidências disponíveis sobre alterações vocais em adultos não intubados com diagnóstico de COVID-19 leve a moderado.

**Estratégia de pesquisa:**

Revisão de escopo conduzida de acordo com as diretrizes PRISMA-ScR, incluindo estudos publicados entre 2019 e 2025. Foram realizadas buscas sistemáticas nas bases MEDLINE (PubMed), EMBASE, LILACS, Scopus, Web of Science e Cochrane Library, além de fontes de literatura cinzenta (Google Scholar, MedRxiv e ProQuest). Descritores controlados e termos livres relacionados à COVID-19 e aos distúrbios da voz foram combinados por meio de operadores booleanos.

**Critérios de seleção:**

Foram incluídos estudos com adultos (18-65 anos) com diagnóstico confirmado de COVID-19 e quadros leves a moderados. Excluíram-se estudos com indivíduos submetidos à intubação endotraqueal, bem como aqueles com histórico prévio de distúrbios vocais ou comorbidades respiratórias. A seleção foi realizada por dois revisores independentes.

**Análise dos dados:**

Os dados foram extraídos e analisados de forma descritiva e quantitativa, considerando características dos estudos, métodos de avaliação vocal e principais desfechos.

**Resultados:**

Dos 35.497 registros identificados, 19 estudos atenderam aos critérios de inclusão. As alterações vocais mais frequentes foram disfonia, rouquidão, redução do tempo máximo de fonação e modificações em medidas acústicas, como jitter, shimmer e índice harmônico-ruído. Sintomas associados incluíram fadiga vocal, tosse, dispneia e desconforto laríngeo, além de impacto negativo na qualidade de vida relacionada à voz.

**Conclusão:**

A COVID-19 leve a moderada pode acarretar comprometimentos vocais clinicamente relevantes, reforçando a necessidade de acompanhamento fonoaudiológico e de pesquisas que subsidiem protocolos de avaliação e reabilitação vocal no período pós-infecção.

## INTRODUÇÃO

A COVID-19, causada pelo vírus SARS-CoV-2, foi declarada pandemia global em março de 2020, afetando milhões de pessoas em todo o mundo^(1)^. Além das manifestações respiratórias agudas, a doença pode desencadear uma tempestade de citocinas, resultando em danos multissistêmicos, incluindo pulmões, coração e trato gastrointestinal^(2)^. Entre os sintomas frequentemente relatados estão alterações sensoriais, como hiposmia, anosmia e disgeusia, bem como manifestações respiratórias que podem impactar diretamente a produção vocal^(3)^.

A produção vocal saudável depende da integração harmoniosa dos sistemas respiratório, fonatório e ressonantal^(4)^. A COVID-19 pode comprometer cada um desses sistemas por diferentes mecanismos, como inflamação laríngea, neuropatias periféricas, fraqueza muscular respiratória e alterações posturais decorrentes de fadiga crônica^(5,6)^, podendo resultar em disfonia, definida como qualquer dificuldade ou alteração na emissão vocal que prejudique a produção natural da voz^(7)^.

Apesar de a COVID-19 apresentar menor relevância clínica em comparação aos anos iniciais da pandemia, o estudo das alterações vocais associadas à infecção permanece relevante. Essas alterações podem integrar a síndrome pós-COVID (COVID longa), reconhecida pela Organização Mundial da Saúde, e representam desafios para reabilitação e acompanhamento clínico^(8)^. Mesmo alterações vocais leves podem gerar impacto funcional significativo, especialmente em indivíduos que dependem da voz para o trabalho, como professores, cantores e profissionais da comunicação^(9,10)^. A escassez de estudos longitudinais que investiguem a duração, a evolução e o manejo terapêutico dessas alterações evidencia uma lacuna na literatura, reforçando a pertinência de uma revisão de escopo^(11)^.

Esta revisão adota o conceito de qualidade vocal como o desempenho funcional da voz, avaliado por meio de medidas perceptivo-auditivas, acústicas e de autorrelato^(7,12,13)^. O fenômeno COVID-19 é abordado em seu espectro completo (fases aguda, pós-aguda e COVID longa)^(8)^, considerando seus mecanismos fisiopatológicos potencialmente impactantes na voz, como inflamação laríngea e neuropatia^(5,6)^. O contexto é delimitado à população de adultos não intubados com quadros leves a moderados, permitindo isolar os efeitos da infecção viral dos danos por intubação e preenchendo uma lacuna importante na literatura, que tradicionalmente prioriza o estudo de casos graves.

Uma busca preliminar em repositórios de protocolos (MEDLINE, Cochrane Database of Systematic Reviews e JBI Evidence Synthesis) não identificou revisões sistemáticas ou de escopo recentes ou em andamento com esse recorte populacional e conceito específico, garantindo a originalidade da revisão.

Diante disso, esta revisão de escopo justifica-se pela necessidade de mapear e sintetizar as evidências disponíveis sobre a qualidade vocal em indivíduos acometidos pela COVID-19, com foco especial em casos leves e moderados, não submetidos à intubação orotraqueal. O objetivo desta revisão de escopo foi mapear as alterações vocais em indivíduos adultos acometidos por COVID-19 não intubados, incluindo casos leves a moderados, considerando alterações perceptivo-auditivas, acústicas e autorrelatadas da qualidade vocal, e situando a análise no contexto clínico de acompanhamento pós-infecção e estudos publicados entre 2019 e 2025, com foco em evidências que possam subsidiar a prática clínica, estratégias de reabilitação vocal e pesquisas futuras.

## MÉTODO

Esta revisão de escopo seguiu recomendações propostas pelo *Preferred Reporting Items for Systematic reviews and Meta-Analyses extension for Scoping Reviews* (PRISMA-ScR) e descritas pelo *Joanna Briggs Institute Reviewer’s Manual* ^(14-17)^. Os objetivos, critérios de inclusão e os métodos utilizados, foram especificados e documentados em um protocolo registrado no Open Science Framework (OSF)^(18)^.

### Critérios de elegibilidade

Para respaldar a elaboração da pergunta de pesquisa e dos critérios de elegibilidade, foi utilizada a estratégia PCC (População, Conceito e Contexto). Foram adotados os seguintes tópicos-chave: a População foram indivíduos com idades entre 18 e 65 anos com diagnóstico de COVID19; o Conceito a qualidade vocal nos indivíduos com diagnóstico de COVID19; e o contexto foram indivíduos com grau leve a moderado da COVID19 tendo como objetivo excluir os indivíduos que foram submetidos a intubação endotraqueal durante a fase aguda da doença. Considerando os tópicos-chave do acrônimo PCC com o objetivo do estudo, foi elaborada a seguinte pergunta de pesquisa: “Quais as características da qualidade vocal em indivíduos adultos com diagnóstico confirmado de COVID-19?”.

### Critérios de inclusão e exclusão

Foram incluídos estudos que apresentassem dados primários ou secundários originais sobre a qualidade vocal na população de interesse, como estudos observacionais, relatos de caso e revisões sistemáticas. Foram excluídos textos que não apresentavam dados originais, como ensaios teóricos, editoriais, cartas e comentários, pois seu foco é a argumentação e opinião, não sendo passíveis de extração de dados para síntese baseada em evidências. Relatos de experiência foram mantidos desde que descrevessem sistematicamente casos ou séries de casos com avaliação vocal. Também foram excluídos estudos que trazem participantes que foram submetidos a intubação endotraqueal durante a internação por COVID-19, apresentassem comorbidades como: fumantes, histórico prévio de distúrbios de voz ou alterações laríngeas, qualquer forma de doença respiratória, dermatológicas, reumáticas ou neurológicas com envolvimento laríngeo; histórico de câncer de cabeça e pescoço ou radioterapia cervical.

### Fontes de pesquisa e estratégia de busca

A busca eletrônica foi realizada em junho de 2024, em seis bases de dados de citações e resumos: Literatura Latino-Americana e do Caribe em Ciências da Saúde (LILACS), Medical Literature Analysis and Retrieval System Online (MEDLINE), EMBASE, Web of science, Scopus, COCHRANE. Foi realizada também a busca em literatura cinzenta em janeiro de 2025: *Google Scholar, MedRxiv, ProQuest.* Além disso, foi realizada busca nas citações dos artigos selecionados. As estratégias de busca foram elaboradas a partir de termos indexados e livres, relacionados à PCC. Utilizaram-se combinações entre os descritores relacionados à COVID-19 (ex.: “COVID-19”, “SARS-CoV-2”) e qualidade vocal (ex.: “Voice”, “Dysphonia”, “Voice Quality”) com operadores booleanos (AND, OR) e truncamentos. A busca foi restrita ao idioma inglês devido à maior cobertura das bases de dados internacionais para o tema investigado. A estratégia completa para cada base de dados está detalhada no Apêndice A.

### Seleção

A seleção dos estudos foi realizada em quatro etapas: (1) calibração (2) triagem de títulos e resumos; (3) exclusão de duplicatas; (4) leitura na íntegra dos artigos elegíveis.

Para a calibração da seleção, foi realizada, inicialmente, a identificação dos estudos nas bases de dados PubMed/MEDLINE e LILACS. Os registros duplicados foram removidos usando ferramentas automáticas do portal *Rayyan*. A calibração foi realizada por dois revisores, de forma independente. Na etapa de calibração foi utilizada uma amostra piloto de 82 artigos. Estes artigos foram avaliados quanto aos critérios de inclusão e exclusão. Foi calculado o coeficiente Kappa de Cohen para medir a concordância interavaliadores, estabelecendo-se um mínimo de 0,7 no coeficiente Kappa de Cohen como critério para avançar para a triagem principal. Após a etapa de calibração, foi realizada a identificação dos estudos nas bases de dados Literatura Latino-Americana e do Caribe em Ciências da Saúde (LILACS), Medical Literature Analysis and Retrieval System Online (MEDLINE), EMBASE, Web of science, Scopus, COCHRANE. Foi realizada também a busca em literatura cinzenta em janeiro de 2025: *Google Scholar, MedRxiv, ProQuest.* Os registros duplicados foram removidos usando ferramentas automáticas do portal *Rayyan.* A seleção foi realizada pelos dois revisores, de forma independente. Na triagem inicial foram aplicados os critérios de inclusão, por meio da leitura do título e resumo, triados manualmente pelos dois revisores. A segunda etapa da seleção consistiu na leitura integral dos artigos, com a aplicação dos critérios de exclusão. Um terceiro pesquisador fez a arbitragem sobre as discordâncias.

### Extração e armazenamento dos dados

A extração de dados foi conduzida de forma padronizada por dois revisores independentes, utilizando planilhas eletrônicas (excel). As variáveis coletadas incluíram: autor, ano e país de publicação; delineamento metodológico; objetivo do estudo; tamanho da amostra; sexo, faixa etária e raça/etnia dos participantes; presença de comorbidades prévias; método de diagnóstico da COVID-19; classificação clínica da doença (leve ou moderada); status vacinal; sinais e sintomas relacionados à COVID-19; protocolos e instrumentos empregados para avaliação vocal; parâmetros acústicos; resultados perceptivo-auditivos; sintomas vocais autorrelatados; tempo de acompanhamento; e principais conclusões relatadas pelos autores.

A síntese foi realizada por meio de análise descritiva e quantitativa, com cálculo de frequências absolutas e relativas das variáveis extraídas. Os resultados foram organizados em quadros e representados graficamente em nuvem de palavras, gráficos de distribuição e mapas geográficos, quando aplicável. Essa abordagem buscou fornecer uma visão abrangente das evidências disponíveis, destacando tendências e lacunas na literatura.

## RESULTADOS

A estratégia de busca nas bases de dados recuperou 35.497 referências, totalizando 442 após a remoção dos duplicados por ferramentas de automação do portal *Rayyan* e a extração manual das referências inelegiveis. Os critérios empregados incluíram a exclusão de referências por tipo de documento, faixa etária incompatível com a população definida (18-65 anos), ausência de menção a COVID-19 confirmada, ano de publicação incompatível com a definida (2019-2025) e idioma da publicação (inglês, português e espanhol). Durante a fase de leitura dos títulos e resumos, 109 publicações foram elegíveis para a leitura na íntegra. Após a leitura, 90 estudos foram excluídos, resultando em 19 publicações incluídas para a síntese (Apêndice B).

### Características dos estudos

Foram incluídos 19 estudos na revisão, com publicações entre 2020 e 2025, sendo o pico em 2021 (n=6) e 2022 (n=5). A maioria dos estudos foi conduzida no Irã (n=7), seguido pelo Turquia (n=2), Egito (n=2) e Brasil (n=2) (Figura 1). Outros países incluídos foram Alemanha, Estados Unidos, Paquistão, Polônia, Suíça e Índia. (Tabela 1)

**Figura 1:** Distribuição de Artigos Incluídos na Revisão de escopo por Nacionalidade

**Legenda:** Mapa mundial destacando a nacionalidade dos artigos incluídos na revisão de escopo. Cada país é representado por uma cor distinta, de acordo com a frequência de publicações: **Azul (Irã):** 7 artigos. **Laranja escuro (Brasil e Egito):** 2 artigos cada. **Vermelho (Alemanha, Estados Unidos, Paquistão, Polônia, Suíça e Índia):** 1 artigo cada

**Tabela 1:** Características dos estudos - parte I

**Autor(es)**	Ano	País
Asiaee et al.^(19)^	2020	Irã
Helding et al.^(9)^	2020	EUA
Eltelety e Nassar^(20)^	2020	Egito
Korkmaz e Güven**^(^** ^21)^	2021	Turquia
Saki et al.^(22)^	2021	Irã
Bartl-Pokorny et al.^(23)^	2021	Alemanha
Tohidast et al.^(24)^	2021	Irã
Dassie-Leite et al.^(25)^	2021	Brasil
Azzam et al.^(26)^	2021	Egito
Gölaç et al.^(12)^	2022	Turquia
Aghadoost et al.^(27)^	2022	Irã
Aghaz et al.^(11)^	2022	Irã
Shanem et al.^(28)^	2022	Paquistão
Khoddami et al.^(13)^	2023	Irã
Saki et al.^(29)^	2023	Irã
Kuć e Michta^(30)^	2023	Polônia
Bueno et al.^(10)^	2024	Brasil
Rahul et al.^(31)^	2024	India
Berti et al.^(32)^	2025	Suíça

Predominaram os estudos observacionais: transversais, analíticos e caso-controle, com variações metodológicas consideráveis. Dois relatos de caso e uma revisão sistemática com meta-análise também foram incluídos. (Tabela 2)

**Tabela 2:** Características dos Estudos - parte II

**Artigo**	**Tipo de pesquisa**	**Objetivo do estudo**	**Tamanho da amostra**	**Número de homens**	**Número de mulheres**	**Faixa etária**
Gölaç et al.^(12)^	observacional, caso-controle	comparar os resultados vocais dos doentes em recuperação com um grupo controle.	80 (40 casos e 40 controles)	40	40	21 a 53 de idade
Korkmaz e Güven**^(^** ^21)^	Relato de caso	apresentar um caso de paralisia de prega vocal unilateral súbita que se acredita estar associado à infecção por COVID-19.	1	0	1	55 anos
Saki et al.^(22)^	estudo observacional, caso-controle	fazer uma avaliação perceptivo-auditiva das características vocais de pacientes com diferentes gravidades da COVID-19.	102	94	38	Grupo de pacientes com média de idade 58,1 ± 9,1 anos Grupo Saudável idade média de 46,7 ± 9,3 anos
Bartl-Pokorny et al.^(23)^	caso controle	fornecer uma visão mais profunda das características da voz de pacientes com COVID-19.	22	14	8	(com COVID: média de idade +/- 60 anos 6,20 anos de desvio padrão; intervalo de idade +/-19-79 anos; sem COVID: idade média +/- 55 anos 6,20 anos de desvio padrão; intervalo de idade +/- 24-85 anos
Khoddami et al.^(13)^	observacional e transversal, caso-controle	determinar a saúde geral e a QV relacionada à voz e investigar a correlação entre eles em pacientes iranianos com COVID-19 e indivíduos saudáveis.	68 (34 controles e 34 casos)	20	14	18 a 50 anos
Saki et al.^(29)^	transversal, caso-controle	comparar sinais e sintomas de fadiga vocal entre pacientes com COVID-19 e pessoas com vozes normais.	60 (30 casos e 30 controle)	18 casos e 14 controle	12 casos e 16 controles	média de idade: 35,69 ± 6,92 anos (controle) idade média: 39,69 ± 10,42 anos (casos)
Aghadoost et al.^(27)^	Transversal, caso-controle	investigar a correlação entre a análise subjetiva e a análise objetiva da voz em pacientes com infecção por COVID-19.	80 (40 casos e 40 controles)	Cada grupo 27 homens	Cada grupo 13 mulheres	Grupo caso: (com média de idade 41,2 ± 5,41 anos). Grupo controle com média de idade 44,50 ± 3,50 anos
Tohidast et al.^(24)^	Caso controle	investigar a qualidade da voz e os sintomas de desconforto do trato vocal em pacientes com COVID-19.	88 pacientes (44 com casos e 44 controle)	24 em cada grupo	20 em cada grupo	A média de idade dos pacientes com COVID-19 e dos indivíduos saudáveis ​​foi de 49,61 +/- 16,48 e 48,52 +/- 13,8 anos, respectivamente.
Dassie-Leite et al.^(25)^	Observacional, analítico, transversal e híbrido	Comparar a ocorrência de sinais e sintomas vocais antes, durante e após a doença do coronavírus (COVID-19) e analisar possíveis fatores de risco para a persistência desses sintomas após a resolução da doença.	45	21	24	média de idade de 44 anos e 10 meses
Asiaee et al.^(19)^	Observacional, caso controle	comparar pacientes com COVID-19 com indivíduos saudáveis para avaliar o efeito desta doença nos parâmetros acústicos.	64 casos e 70 controles (134)	38 casos e 33 controle	26 casos e 37 controle	Média de 52,3 anos no caso e 42,35 anos nos controles
Helding et al.^(9)^	Artigo de revisão/discussão	Discutir as sequelas da COVID‐19 e seus impactos na voz, com foco em cantores.	-	-	-	-
Aghaz et al.^(11)^	revisão sistemática e meta-análise	estimar a prevalência de disfonia em pacientes com COVID-19.	1410	28,20%	32,80%	42,71 ano
Azzam et al.^(26)^	Estudo clínico	Detectar a ocorrência de sintomas vocais em pacientes com COVID-19 no Egito e investigar os achados videolaringoscópicos associados.	106	28	78	18 a 66 anos
Bueno et al.^(10)^	Estudo observacional	Identificar deficiências vocais e mudanças na qualidade de vida relacionada à voz em pacientes com deficiência pulmonar associada à COVID-19.	35	22	13	18 a 65 anos
Shanem et al.^(28)^	Estudo transversal retrospectivo	Investigar o impacto da COVID-19 na função das cordas vocais e na produção de voz.	379	168	211	20 a 50 anos
Kuć e Michta^(30)^	Estudo observacional	diagnosticar os tipos de distúrbios da fala que ocorrem em sobreviventes da COVID-19.	30	15	15	30 a 60 anos
Berti et al.^(32)^	Estudo comparativo	Comparar as características de voz e fala entre indivíduos pós-COVID-19 e indivíduos de controle.	134 (70 controle e 64 casos)	30 controles e 25 casos	40 controles e 39 casos	40 a 60 anos
Eltelety e Nassar^(20)^	estudo de caso	Relatar um paciente com COVID-19 apresentando principalmente rouquidão da voz e anosmia.	1	1	0	27
Rahul et al.^(31)^	Caso controle	Explorar as mudanças perceptivas, os resultados autorrelatados e as mudanças acústicas na voz de indivíduos infectados pela COVID-19 imediatamente após o período de isolamento e compará-los com o grupo controle.	25	0	25	18 a 40 anos

Os estudos tiveram como objetivo principal investigar os efeitos da COVID-19 na qualidade vocal por meio de diferentes avaliações, incluindo análise acústica^(12,19,20,23,27)^, julgamento perceptivo-auditivo^(22,24)^, protocolos de autopercepção^(13,25,29)^ e exames instrumentais^(13,31)^. Diversos trabalhos^(10,12,27,31)^ compararam indivíduos com COVID-19 a grupos controle saudáveis, buscando identificar alterações vocais, sintomas persistentes, fadiga vocal e impacto na qualidade de vida relacionada à voz. Também foram exploradas correlações entre medidas subjetivas e objetivas da voz, além de relatos de casos clínicos específicos^(20,21)^, como paralisia vocal súbita e manifestações leves como rouquidão. Alguns estudos^(9,10)^ destacaram populações específicas, como cantores ou pacientes com comprometimento pulmonar, ampliando a compreensão sobre as possíveis sequelas vocais associadas à infecção. (Tabela 2)

Os tamanhos amostrais variaram de 1 (relato de caso) a 1.410 participantes (meta-análise) com faixa etária de 18 a 66 anos, sendo a maioria do gênero masculino. (Tabela 2)

Alguns estudos utilizaram somente o teste de reação em cadeia da polimerase com transcrição reversa (RT-PCR) realizado em laboratório^(10,12,21,22,25,27,29,30)^. Outros estudos fizeram o teste RT-PCR associado a tomografía computadorizada (TC)^(13,19,24,28,33)^ ou Teste Rápido de Antígeno (TR-Ag) ou Autoteste de Antígeno (AT-Ag)^(20)^.

### Contexto clínico encontrados nos estudos

Os sinais e sintomas da COVID-19 relatados nos estudos evidenciam uma variedade de manifestações clínicas, com predominância dos sintomas respiratórios (Tabela 3). Tosse seca, dispnéia e desconforto respiratório foram os mais frequentemente descritos, aparecendo de forma isolada ou em associação a pneumonia leve a moderada^(10,12,19,27)^. Febre e fadiga também foram amplamente mencionadas, sendo a fadiga uma queixa recorrente tanto na fase aguda quanto entre pacientes com sintomas persistentes^(25,29)^. Sintomas neurossensoriais, como anosmia e disgeusia, estiveram presentes em vários relatos, além de cefaleia, mialgia e dores articulares^(13,20,24)^. Em alguns estudos, a presença de alterações vocais como rouquidão, disfonia e fadiga vocal destacou-se, aparecendo tanto como sintoma isolado quanto em associação aos sintomas respiratórios, chegando a ser o principal indicador de complicações laríngeas, como a paralisia de prega vocal^(21,31)^. Outros sintomas vocais incluíram garganta seca, pigarro e sensação de muco espesso^(10,24)^.

**Tabela 3:** Contexto clínico encontrados nos estudos

**Artigo**	**Classificação da COVID-19**	**diferentes *status* vacinais**	**Sinais e sintomas da COVID-19**	**Sinais e sintomas vocais**
Gölaç et al.^(12)^	O artigo não classifica a COVID-19	não menciona	tosse, dificuldade em respirar, cansaço, dificuldade em falar e dor	Tosse, dificuldade em respirar, cansaço, dificuldade em falar e dor
Korkmaz e Güven**^(^** ^21)^	O artigo não classifica a COVID-19	foi vacinado contra a COVID-19 com duas doses da vacina antes de ser diagnosticado com a infecção.	Tosse e falta de ar, Febre, Fadiga, rouquidão progressiva.	Rouquidão (Disfonia), Dificuldade para Falar, Fadiga Vocal
Saki et al.^(22)^	Leve (Mild), Moderada (Moderate), Grave (Severe)	não menciona	não forneceu	Disfonia, Rouquidão, dificuldades para controlar a emissão vocal
Bartl-Pokorny et al.^(23)^	Os participantes do COVID apresentavam sintomas respiratórios leve a moderado.	não menciona	Sintomas respiratórios leve a moderados.	não há relato
Khoddami et al.^(13)^	leve a moderada	não relatado	Ausência de pneumonia e complicações leves até Febre e sinais de pneumonia e infecção.	não especificado
Saki et al.^(29)^	não classifica	não especifica	Tosse, faringite, dor de garganta e dificuldade respiratória.	Fadiga Vocal, Voz áspera, soprosa, esforço vocal e quebras de voz.
Aghadoost et al.^(27)^	COVID-19 leve a moderada	não menciona	pneumonia, febre e outros sintomas respiratórios, mas sem insuficiência respiratória que exigisse ventilação mecânica.	Disfonia (alterações na voz, como rouquidão), Redução na qualidade vocal, com aumento de rugosidade, soprosidade, tensão e esforço vocal, Redução no tempo máximo de fonação
Tohidast et al.^(24)^	Leve, moderado e grave (UTI)	Não informado	não informado	Desconforto vocal (queimação, dor, secura, irritabilidade e caroço na garganta, aperto)
Dassie-Leite et al.^(25)^	Leve, aguda, grave, crítica	não informado	tosse, febre, dispneia, sintomas musculoesqueléticos (mialgia, dor nas articulações, fadiga), sintomas gastrointestinais, anosmia e disgeusia.	à sensibilidade laríngea e alterações vocais, Disfonia,
Asiaee et al.^(19)^	não informado	não informado	não informado	não especificado
Helding et al.^(9)^	não aplicável	não aplicável	sintomas respiratórios, neurológicos e pós-infecciosos	Revisão de evidências
Aghaz et al.^(11)^	não informado	não informado	sinais e sintomas específicos não são detalhados	disfonia
Azzam et al.^(26)^	leve a moderada	não informado	disfonia, fonastenia, obstrução nasal, perda do olfato, disfunção do paladar, dor de garganta, muco pegajoso e disfagia	Disfonia e fonastenia
Bueno et al.^(10)^	não informado	não informado	dispneia e tosse	disfonia, fadiga vocal e laringoespasmo
Shanem et al.^(28)^	não informado	não informado	não informado	Voz fraca e trêmula, voz pouco nítida e alterações vocais
Kuć e Michta^(30)^	não informado	não informado	não informado	rouquidão, tosse, dor ao engolir e sensação de corpo estranho na garganta
Berti et al.^(32)^	leves a moderados	não informado	coriza, dor de garganta, tosse, febre, dor de cabeça, perda de paladar/olfato, fadiga, dores musculares e/ou diarreia	não informado
Eltelety e Nassar^(20)^	não informado	não informado	Rouquidão da voz e anosmia	Rouquidão da voz
Rahul et al.^(31)^	não informado	não informado	Tosse, garganta seca e pigarro	dificuldade em aumentar o volume e outros problemas vocais

Os sinais e sintomas vocais associados à COVID-19, conforme relatados nos estudos, englobam uma variedade de alterações perceptivas, acústicas e autoperceptivas (Tabela 3). A disfonia, frequentemente descrita como rouquidão, foi o sintoma vocal mais recorrente e, em alguns casos, a principal queixa que motivou a investigação clínica, sendo inclusive o primeiro sintoma percebido por alguns participantes^(11,12,21,23)^. Além da rouquidão, outros sintomas relatados incluíram dificuldade para falar de forma clara, voz fraca, trêmula, com esforço, soprosidade, aspereza, tensão vocal, alterações na intensidade vocal, e na qualidade geral da voz^(24,27,28,31)^. Sintomas como fadiga vocal, dor, queimação, secura, sensação de corpo estranho ou aperto na garganta também foram destacados, evidenciando um desconforto laríngeo persistente^(10,24,29)^.

A Figura 2 mostra uma Nuvem de palavras que destaca os sinais e sintomas vocais mais frequentes associados à COVID-19, evidenciando termos como rouquidão, fadiga vocal e alterações na voz.

**Figura 2:** Sinais e sintomas vocais associados a COVID-19

**Legenda:** Mostra os principais sintomas vocais relatados nos estudos analisados, como disfonia, rouquidão, fadiga vocal, redução do tempo máximo de fonação (TMF), e presença de paralisia de prega vocal

### Avaliações vocais encontradas nos estudos

Os estudos incluídos utilizaram uma variedade de métodos e instrumentos para avaliar a função vocal de indivíduos acometidos pela COVID-19, predominantemente: análises acústicas, julgamentos perceptivo-auditivos e autopercepção (Figura 3). No campo da análise acústica, destacaram-se medidas como o Tempo Máximo de Fonação (TMF), *jitter*, *shimmer*, frequência fundamental (Fo), intensidade mínima e índice harmônico-ruído (HNR)^(19,23,27,31)^. Alguns estudos calcularam o *Dysphonia Severity Index (DSI)* como uma medida composta da gravidade da disfonia^(12,27)^ (Figura 3). O Julgamento perceptivo-auditivo foi conduzido principalmente por meio dos protocolos CAPE-V e GRBAS, incluindo tarefas de emissão sustentada de vogais, leitura de frases e fala espontânea^(24,29)^. A escala CAPE-V foi aplicada em diferentes idiomas e contextos, com foco em parâmetros como aspereza, soprosidade, tensão e severidade geral da voz^(12,27)^ (Figura 3).

**Figura 3:** Frequência dos protocolos de avaliação vocal utilizados

**Legenda:** Ilustra os questionários e protocolos mais utilizados para avaliação da qualidade de vida relacionada à voz: VHI-10 (Voice Handicap Index-10), V-RQOL (Voice-Related Quality of Life), VFI (Índice de Fadiga Vocal) e VTDS (Vocal Tract Discomfort Scale)

Na esfera dos instrumentos autorrelatados, os questionários mais utilizados foram o *Voice Handicap Index (VHI-10)* ^(10,12,13)^, que avalia o impacto da disfonia na vida diária, e o *Voice-Related Quality of Life (V-RQOL)* ^(13,25)^, que mede a qualidade de vida relacionada à voz. Também foram utilizados o *Índice de Fadiga Vocal (VFI)* ^(24,29)^ e o *Vocal Tract Discomfort Scale (VTD)* ^(24,25)^, além da *SSL* (lista de sinais e sintomas vocais)^(25)^ em diferentes momentos da infecção (pré, durante e pós-COVID) (Figura 3). Complementarmente, alguns estudos^(20,21,26)^ incluíram exames como a laringoscopia ou ultrassonografia laríngea para investigar a anatomia e mobilidade das pregas vocais, especialmente em casos de paralisia laríngea. Embora nem todos os estudos tenham especificado os protocolos utilizados, de forma geral, houve uma tendência em combinar diferentes métodos para fornecer uma avaliação vocal mais abrangente e sensível aos efeitos da COVID-19.

A análise acústica foi amplamente utilizada nos estudos revisados (Tabela 4) para avaliar objetivamente alterações na qualidade vocal de indivíduos que se recuperaram da COVID-19. Diversos parâmetros foram medidos por meio de softwares como *Praat* ^(12,19,32)^ e *VoiceSauce* ^(19)^, utilizando tanto fonação sustentada quanto fala espontânea.

**Tabela 4:** Avaliações vocais encontradas nos estudos

**Artigo**	**Avaliação vocal**	**Análise acústica**
Gölaç et al.^(12)^	Análise Acústica	Parâmetros Acústicos Avaliados:
	Medida aerodinâmica: TMF	Frequência Fundamental Média (F0)
	Questionários de autopercepção: Voice Handicap Index-10 (VHI-10), Voice-Related Quality of Life (V-RQOL)	Jitter Local
	Julgamento perceptivo auditivo: CAPE-V	Shimmer Local
		Razão Harmônico-Ruído (HNR)
		Proeminência do Pico Cepstral (CPP).
		Métodos e Ferramentas Utilizados:
		Software: As análises acústicas foram realizadas utilizando o software Praat (versão 6.0.17)
		Amostras de Voz: As amostras de voz foram coletadas por meio da fonação sustentada da vogal /a/ e da fala conectada (terceira frase do protocolo CAPE-V em turco).
		Segmento Analisado: Para as medidas de perturbação (jitter, shimmer e HNR), foi utilizado o segmento médio da vogal /a/ na parte mais estável da fonação. Para o CPP, foi analisada a terceira frase do protocolo CAPE-V.
Korkmaz e Güven**^(^** ^21)^	Análise acústica	Frequência Fundamental (F0): A frequência fundamental, ou pitch, foi medida para verificar a variação no tom da voz, já que a paralisia unilateral pode afetar a simetria do movimento das cordas vocais e, consequentemente, a frequência de emissão vocal.
	avaliação do índice de desvantagem vocal (VHI)	Jitter: Jitter refere-se à variação na frequência fundamental da voz e pode ser utilizado para avaliar a estabilidade do tom vocal. A paralisia pode causar flutuações no tom, refletindo na irregularidade vocal.
	Laringoscopia: A laringoscopia direta foi realizada para examinar as cordas vocais e identificar a paralisia unilateral.	Shimmer: O shimmer mede a variação na amplitude do som, indicando a regularidade da intensidade vocal. A paralisia das cordas vocais pode afetar a consistência da produção vocal, alterando o shimmer.
	Medida aerodinâmica: TMF, utilizando a relação s/z.	Índice de Qualidade Vocal: Parâmetros como a regularidade do som (medidos pelo jitter e shimmer) são frequentemente usados para avaliar a qualidade vocal. No caso de paralisia vocal, esses parâmetros podem ser alterados, refletindo uma qualidade vocal comprometida.
Saki et al.^(22)^	Julgamento perceptivo auditivo: GRBAS	A análise acústica foi realizada para avaliar parâmetros como frequência fundamental (F0), intensidade e perturbações na frequência e amplitude da voz, proporcionando uma avaliação mais objetiva das alterações
Bartl-Pokorny et al.^(23)^	analisou gravações de vogais sustentadas (/i:/, /e:/, /o:/, /u:/ e /a:/) produzidas por participantes falantes de alemão. A pesquisa se concentrou em 88 características acústicas extraídas dessas gravações, sem especificar protocolos de avaliação vocal tradicionais, como a duração máxima de fonação ou a intensidade vocal. A análise estatística foi realizada utilizando o teste U de Mann-Whitney e o cálculo de tamanhos de efeito para identificar diferenças significativas entre os grupos de participantes.	A análise acústica incluiu os seguintes parâmetros:
		Frequência fundamental (F0): A frequência média da voz, que pode refletir mudanças no controle vocal e nas tensões nas cordas vocais.
		Razão harmônico-para-ruído (HNR): Uma medida que indica a clareza da voz. Quanto mais baixa a razão HNR, maior a presença de ruído na voz.
		Coeficientes cepstrais de Mel (MFCC): Características espectrais que ajudam a descrever o conteúdo acústico da voz de uma forma que simula a percepção auditiva humana.
		Inclinação espectral: Reflete como a energia se distribui nas frequências do som vocal.
		Duração da vogal e número de segmentos sonoros por segundo: Indicadores do ritmo e fluidez da produção vocal.
Khoddami et al.^(13)^	Voice Handicap Index (VHI): Este índice é uma ferramenta amplamente utilizada para avaliar o impacto da disfunção vocal na qualidade de vida dos indivíduos. Ele ajuda a medir o grau de desconforto e limitação que os problemas vocais causam na vida cotidiana dos pacientes.	não menciona a realização de análises acústicas da voz dos participantes
	Avaliação da qualidade de vida relacionada à saúde: Embora o estudo não forneça detalhes específicos sobre o protocolo exato usado para medir a qualidade de vida, é mencionado que a avaliação da qualidade de vida foi uma parte importante da análise, comparando os pacientes recuperados de COVID-19 com os sujeitos saudáveis.	
Saki et al.^(29)^	Avaliação Acústica da Voz	Parâmetros Avaliados: Jitter (%), Shimmer (%), MPT (segundos), HNR (dB).
	Julgamento perceptivo auditivo: CAPE-V	Resultados:
	Questionário de autopercepção: Índice de Fadiga Vocal	Pacientes com COVID-19 apresentaram maior Jitter, Shimmer e menor HNR e MPT em comparação com o grupo controle.
		O uso prolongado da voz (após a leitura do texto) exacerbou as irregularidades vocais nos pacientes com COVID-19.
		Correlações:
		Os parâmetros acústicos (Jitter, Shimmer, HNR) correlacionaram-se significativamente com a fadiga vocal (cansaço da voz e desconforto físico).
Aghadoost et al.^(27)^	Julgamento perceptivo auditivo: CAPE-V	Perturbação de Frequência (Jitter):Frequência Fundamental mais Alta (F0 alta):
	Questionário de autopercepção: Dysphonia Severity Index (DSI)	
Tohidast et al.^(24)^	Julgamento perceptivo auditivo: GRBAS	não foi realizada
	Questionário de autopercepção: VTD (desconforto do trato vocal)	
Dassie-Leite et al.^(25)^	lista de sinais e sintomas vocais (SSL), que investiga a presença ou	não realizada
	ausência de 14 sinais ou sintomas vocais. Foi utilizada uma tradução do instrumento para o português brasileiro. Cada sintoma foi abordado em relação a três momentos distintos:	
	antes, durante e depois da COVID-19.	
Asiaee et al.^(19)^	Analise acústica	Um script Praat foi
		usado para extrair os valores locais de jitter, shimmer, MPT e
		o número de quebras de voz. As medições foram realizadas usando as configurações padrão no Praat. Frequência fundamental, CPP, HNRs e (H1-H2) foram automaticamente
		extraídos usando o VoiceSauce,37 um freeware para análise de voz.
		A medição de F0 foi feita usando o algoritmo padrão do
		VoiceSauce, ou seja, STRAIGHT. No VoiceSauce, o CPP é calculado usando o algoritmo
		proposto por Hillenbrand et al.14 Os valores de HNR são medidos
		usando uma janela variável de cinco períodos de pitch pelo algoritmo de Krom.39 HNR05, HNR15, HNR25, HNR35 medem
		HNR de 0-500 Hz, 0-1500 Hz, 0-2500 Hz e 0-3500 Hz,
		respectivamente. Este estudo usou HNR35 (doravante HNR).
		Finalmente, H1*-H2* foi usado para avaliação (H1-H2), que
		é o H1-H2 corrigido para o efeito de formantes com base no
		algoritmo proposto por Iseli et al40 e usado no Voice- Sauce.
Helding et al.^(9)^	Revisão de estudos com métodos acústicos e perceptuais	Não aplicável
Aghaz et al.^(11)^	Os contextos não fornecem protocolos específicos de avaliação vocal usados nos estudos	Os contextos não fornecem detalhes sobre nenhuma análise acústica realizada.
Azzam et al.^(26)^	Inclui avaliação perceptivo-auditiva da voz e exame videolaringoscópico	Não mencionada nos contextos fornecidos.
Bueno et al.^(10)^	Índice de Desvantagem Vocal 10 (IDV-10) e o protocolo de Qualidade de Vida Relacionada à Voz (QVV) e TMF	Não especificada nos contextos fornecidos.
Shanem et al.^(28)^	O questionário Voice Handicap Index (VHI) foi usado para avaliar as preocupações com a voz	não foi realizada
Kuć e Michta^(30)^	incluíram gravações de fala espontânea, repetição de palavras e frases, um monólogo e sequências automatizadas de palavras; a avaliação perceptiva foi conduzida usando a escala GRBAS	Não especificada nos contextos fornecidos
Berti et al.^(32)^	As gravações foram feitas usando o software SPIRA com base em três tarefas verbais: produção sustentada da vogal /a/, leitura de uma frase e produção de rima	Conduzida com o software PRAAT, com foco em vários parâmetros acústicos
Eltelety e Nassar^(20)^	A ultrassonografia laríngea transcervical de acompanhamento foi realizada para avaliar a mobilidade das pregas vocais	Não especificado
Rahul et al.^(31)^	Avaliação perceptiva, resultados autorrelatados usando o Voice Handicap Index (VHI-10) e análise acústica foram conduzidos	O estudo revelou diferenças significativas nos parâmetros acústicos entre os grupos experimental e controle

A revisão aponta que a infecção por COVID-19 pode impactar negativamente a qualidade vocal, mesmo após a fase aguda da doença^(12,25)^. Alterações perceptivo-auditivas, como rugosidade, tensão e alteração global da qualidade vocal, foram frequentemente observadas, além de prejuízos em medidas de autopercepção, com escores elevados em instrumentos como VHI-10 e V-RQOL^(13,24,25,27)^. Embora muitos estudos não tenham encontrado diferenças estatisticamente significativas em parâmetros acústicos como Fo, jitter e shimmer, foi comum a redução do TMF^(19,23)^. A presença de sintomas como tosse, dispneia e fadiga durante a COVID-19 se correlacionou com maior impacto vocal percebido^(12,29)^. Casos de paralisia de prega vocal e alterações neurológicas também foram relatados, sugerindo possíveis mecanismos centrais e periféricos envolvidos nas disfunções vocais pós-COVID^(20,21)^.

Para a síntese, os dados foram organizados por características dos estudos, tipo de avaliação vocal e contexto clínico, considerando a variabilidade metodológica dos estudos e destacando evidências consistentes e discrepantes.

## DISCUSSÃO

As 19 publicações incluídas neste estudo mostram que a COVID-19 está associada a alterações vocais significativas, mesmo em casos leves a moderados e que não foram submetidos a intubação endotraqueal. Os achados corroboram com estudos anteriores que destacam o impacto multifatorial do SARS-CoV-2 nos sistemas respiratório, fonatório e neurológico, resultando em disfunções vocais ^(5,6,29)^.

Sete estudos (36,8%) incluídos na revisão foram realizados na República Islâmica do Irã. De acordo com a Organização Mundial da Saúde (OMS), até 28 de fevereiro de 2022, o país havia registrado mais de 6,6 milhões de casos confirmados de COVID-19 e aproximadamente 140 mil mortes^(33)^. Durante a pandemia, o Irã enfrentou limitações significativas em sua capacidade de realizar sequenciamento genômico, o que dificultou a vigilância epidemiológica e o rastreamento de novas variantes do SARS-CoV-2, impactando negativamente a efetividade da resposta nacional à crise sanitária. Em resposta a essas limitações, a OMS e seus parceiros internacionais forneceram apoio técnico e financeiro ao país, promovendo a aquisição de equipamentos com tecnologia avançada, *softwares* específicos e capacitações técnicas. Além disso, campanhas abrangentes de comunicação de risco e estratégias de engajamento comunitário foram implementadas com o objetivo de fomentar uma resposta centrada nas comunidades, estimulando a procura por testagem, tratamento e vacinação^(33)^.

Os estudos incluídos totalizaram 2.730 participantes com predominância do gênero masculino. Apesar da relevância da variável raça/cor da pele para a compreensão das desigualdades em saúde, observou-se que nenhum dos estudos incluídos realizou análise por raça ou etnia. Essa ausência de dados limita a análise de possíveis disparidades raciais no impacto vocal da COVID-19. A literatura tem demonstrado que grupos racialmente minorizados foram mais afetados pela pandemia em termos de infecção, gravidade e acesso aos cuidados de saúde, em parte devido a fatores estruturais como desigualdade socioeconômica, condições de trabalho e menor acesso à reabilitação pós-COVID^(34)^. A não inclusão sistemática da variável raça nos estudos sobre qualidade vocal impede, portanto, a identificação de populações potencialmente mais vulneráveis.

A disfonia foi o sintoma mais comumente descrito, com frequência relatada variando de 25% a 79%, dependendo da amostra e dos critérios adotados nos estudos incluídos^(8,11,12)^. A metanálise conduzida por Aghaz et al.^(11)^ apontou uma prevalência combinada de 25,1% durante a fase aguda da doença, com persistência de sintomas vocais em 17,1% após a recuperação. Outros estudos relataram percentuais ainda mais elevados, sugerindo que os métodos de avaliação e as características clínicas das coortes influenciam substancialmente os achados^(8).^ Observou-se, também, uma tendência maior de prevalência de disfonia entre participantes do gênero feminino, conforme apontado na meta-análise de Aghaz et al.^(11)^ e em estudos observacionais incluídos nesta revisão.^(10,13,25)^ Esses dados sugerem que, além da frequência de sintomas, o impacto funcional da disfonia pode ser mais acentuado no público do gênero feminino. Essa diferença pode ser atribuída a fatores anatômicos e hormonais — como pregas vocais mais delgadas e vibração glótica mais rápida — que tornam a laringe feminina potencialmente mais vulnerável a alterações. Além disso, aspectos comportamentais e sociais podem levar o público do gênero feminino a perceberem e relatarem com mais frequência sintomas vocais, refletindo maior sensibilidade às mudanças na qualidade da voz.

A análise acústica revelou aumento no jitter, shimmer e redução no TMF, indicando instabilidade vocal e possível comprometimento respiratório^(19,23)^. Asiaee et al.^(19)^ encontraram diferença estatisticamente significativa nos parâmetros acústicos entre indivíduos recuperados da COVID-19 e participantes do grupo controle, compostos por indivíduos saudáveis sem histórico da doença. Além disso, o tempo máximo de fonação (TMF), uma medida da eficiência respiratória e da competência glótica, esteve sistematicamente reduzido nos grupos acometidos pela infecção^(10,12,25)^. Gölaç et al.^(12)^ verificaram TMF médio de 11,9 segundos nos pacientes pós-COVID-19, em contraste com 16,4 segundos nos controles saudáveis. De forma semelhante, Bueno et al.^(10)^ observaram TMF de 8,9 segundos nos pacientes com pneumonia grave e 10,7 segundos naqueles com pneumonia moderada, frente a 16,2 segundos nos controles saudáveis^(10)^. Tais achados corroboram a hipótese de que a COVID-19 pode comprometer a laringe e o suporte respiratório necessário à produção vocal. Além disso, a redução na relação harmônico-ruído (HNR) sugere maior presença de ruído na voz, associada a inflamação laríngea ou fraqueza muscular^(20)^. Os participantes relataram fadiga vocal persistente, dificuldade em projetar a voz e sensação de esforço ao falar^(22,25)^.

Instrumentos como o Voice Handicap Index (VHI-10) e o Voice-Related Quality of Life (V-RQOL) confirmaram que essas alterações impactam negativamente a qualidade de vida, especialmente em profissionais da voz^(12,13)^. Isso evidencia que, mesmo nos casos em que a disfonia é leve do ponto de vista clínico, ela pode ter impacto funcional importante, afetando a comunicação cotidiana e a qualidade de vida, especialmente em indivíduos que dependem da voz como ferramenta de trabalho.

As alterações vocais podem ser atribuídas a múltiplos fatores com comprometimento respiratório: redução do TMF e a voz fraca estão associadas à diminuição da capacidade pulmonar pós-COVID-19^(28)^; inflamação laríngea: tosse persistente e refluxo gastroesofágico secundário à infecção podem causar edema e irritação das pregas vocais^(31)^; Neuropatia periférica: casos de paralisia de prega vocal^(21)^ e alterações na inervação laríngea sugerem que o SARS-CoV-2 pode afetar nervos cranianos; e fadiga muscular generalizada: a síndrome pós-COVID-19 pode levar à fraqueza dos músculos respiratórios e laríngeos, afetando a projeção vocal^(9)^.

Outros sintomas frequentemente relatados incluíram garganta seca, dor ou queimação na garganta, sensação de muco espesso e fadiga vocal^(10,24,29)^. Em estudos que utilizaram a Vocal Tract Discomfort Scale (VTDS), a sensação de secura destacou-se com altos escores e tamanho de efeito elevado, sugerindo que alterações na lubrificação ou inflamação laríngea são componentes relevantes da experiência subjetiva de disfonia^(28)^.

Os resultados destacam a necessidade de Avaliação vocal sistemática em pacientes recuperados da COVID-19, utilizando protocolos como CAPE-V, GRBAS e análises acústicas^(24,27)^; Intervenção precoce, incluindo fonoterapia para reabilitação respiratória e vocal, especialmente em casos de disfonia persistente^(32)^; e acompanhamento multidisciplinar, envolvendo otorrinolaringologistas, fonoaudiólogos e pneumologistas, para manejar sequelas integradas^(30)^.

As manifestações vocais persistentes observadas em alguns participantes sugerem uma possível vinculação à síndrome pós-COVID, também chamada de COVID longa. A OMS^(8)^ reconhece como critério clínico a presença de sintomas prolongados além de 12 semanas após o início da infecção e a disfonia pode figurar entre essas queixas duradouras, como observado em estudos incluídos nesta revisão^(10,24,25,29)^.

A heterogeneidade metodológica entre os estudos — incluindo protocolos de avaliação vocal, instrumentos utilizados, critérios de inclusão e períodos de seguimento — limita a generalização dos resultados. A maioria dos estudos apresentou acompanhamento de curto prazo e amostras restritas, dificultando a análise longitudinal das alterações vocais.

Quanto à revisão, não foi realizada avaliação formal da qualidade metodológica dos estudos incluídos, o que constitui uma limitação. Além disso, vieses potenciais na busca e seleção dos estudos, bem como a exclusão de artigos em idiomas diferentes do inglês, português e espanhol, podem ter influenciado os achados.

Os resultados reforçam a importância de avaliação vocal sistemática em pacientes pós-COVID-19, utilizando protocolos padronizados (CAPE-V, GRBAS, análises acústicas) e instrumentos de autorrelato. A intervenção precoce, incluindo fonoterapia para reabilitação respiratória e vocal, e o acompanhamento multidisciplinar são recomendados para mitigar sequelas funcionais.

Futuros estudos devem ser longitudinais, multicêntricos e com amostras maiores, incluindo análises por sexo, raça/etnia e variáveis clínicas relevantes, a fim de compreender a evolução das alterações vocais, mecanismos fisiopatológicos e estratégias eficazes de reabilitação. Além disso, protocolos padronizados podem reduzir a heterogeneidade metodológica e facilitar comparações entre diferentes populações e contextos clínicos.

## CONCLUSÃO

Esta revisão de escopo evidencia que a COVID-19 está associada a alterações vocais objetivas e subjetivas, com impacto funcional e na qualidade de vida.

Este estudo reforça a importância da voz como marcador funcional na COVID-19 e a necessidade de abordagem multidisciplinar para o manejo desses pacientes.

Recomenda-se novos estudos longitudinais para avaliar a duração e a reabilitação nos casos de alterações vocais pós-COVID-19.

## Apêndice A Estratégia de busca

**Table:**

Base de dados	Data	Estratégia de busca	Número de artigos
MEDLINE/PUBMED	junho/2024	((“COVID-19”[Mesh] OR “2019 Novel Coronavirus Disease”[tiab] OR “2019 Novel Coronavirus Infection”[tiab] OR “2019-nCoV Disease”[tiab] OR “2019-nCoV Infection”[tiab] OR “COVID19 Coronavirus Disease “[tiab] OR “Coronavirus Disease-19”[tiab] OR “SARS Coronavirus 2 Infection”[tiab] OR “SARS-CoV-2 Infection”[tiab] OR “Severe Acute Respiratory Syndrome Coronavirus 2 Infection”[tiab] OR “Covid 19”[tiab] OR “covid-19”[tiab] OR “covid19”[tiab] OR “coronavirus”[tiab] OR “SARS-COV-2”[tiab] OR “SARS-COV2” [tiab] OR “COVID disease”[tiab] OR “COVID sequelae”[tiab] OR ”COVID complications”[tiab] OR “COVID survivors”[tiab] OR “COVID infection” [tiab] OR “COVID infections”[tiab]) AND (“Voice Quality”[Mesh] OR “Voice”[Mesh] OR “Voice Qualities”[tiab] OR “Voices”[tiab] OR “Voice Disorders“[tiab] OR “Voice Disturbance “[tiab] OR “Voice Disturbances “[tiab] OR “Voice”[tiab] OR “Vocal quality”[tiab] OR “Voice quality”[tiab] OR “vocal health”[tiab] OR “voice health”[tiab] OR “vocal disorder”[tiab] OR “voice production”[tiab] OR “voice pathology”[tiab] OR “voice dysfunction”[tiab] OR “vocal dysfunction”[tiab]))	1509
EMBASE	junho/2024	('covid 19'/exp OR '2019 novel coronavirus disease':ti,ab OR '2019 novel coronavirus infection':ti,ab OR '2019-ncov disease':ti,ab OR '2019-ncov infection':ti,ab OR 'covid19 coronavirus disease':ti,ab OR 'coronavirus disease-19':ti,ab OR 'sars coronavirus 2 infection':ti,ab OR 'sars-cov-2 infection':ti,ab OR 'severe acute respiratory syndrome coronavirus 2 infection':ti,ab OR 'covid 19':ti,ab OR 'covid-19':ti,ab OR 'covid19':ti,ab OR 'coronavirus':ti,ab OR 'sars-cov-2':ti,ab OR 'sars-cov2':ti,ab OR 'covid disease':ti,ab OR 'covid sequelae':ti,ab OR 'covid complications':ti,ab OR 'covid survivors':ti,ab OR 'covid infection':ti,ab OR 'covid infections':ti,ab) AND ('voice quality'/exp OR 'voice'/exp OR 'voice qualities':ti,ab OR 'voices':ti,ab OR 'voice disorders':ti,ab OR 'voice disturbance':ti,ab OR 'voice disturbances':ti,ab OR 'voice':ti,ab OR 'vocal quality':ti,ab OR 'voice quality':ti,ab OR 'vocal health':ti,ab OR 'voice health':ti,ab OR 'vocal disorder':ti,ab OR 'voice production':ti,ab OR 'voice pathology':ti,ab OR 'voice dysfunction':ti,ab OR 'vocal dysfunction':ti,ab)	1765
LILACS	junho/2024	((mh:(“COVID-19” OR “Voice Quality” OR “Voice”)) OR (tw:(“2019 Novel Coronavirus Disease” OR “2019 Novel Coronavirus Infection” OR “2019-nCoV Disease” OR “2019-nCoV Infection” OR “COVID19 Coronavirus Disease” OR “Coronavirus Disease-19” OR “SARS Coronavirus 2 Infection” OR “SARS-CoV-2 Infection” OR “Severe Acute Respiratory Syndrome Coronavirus 2 Infection” OR “Covid 19” OR “covid-19” OR “covid19” OR “coronavirus” OR “SARS-COV-2” OR “SARS-COV2” OR “COVID disease” OR “COVID sequelae” OR “COVID complications” OR “COVID survivors” OR “COVID infection” OR “COVID infections”))) AND ((mh:(“Voice Quality” OR “Voice”)) OR (tw:(“Voice Qualities” OR “Voices” OR “Voice Disorders” OR “Voice Disturbance” OR “Voice Disturbances” OR “Voice” OR “Vocal quality” OR “Voice quality” OR “vocal health” OR “voice health” OR “vocal disorder” OR “voice production” OR “voice pathology” OR “voice dysfunction” OR “vocal dysfunction”)))	279
SCOPUS	junho/2024	((INDEXTERMS (“COVID-19”) OR INDEXTERMS (“Voice Quality”) OR INDEXTERMS (“Voice”)) OR (TITLE-ABS-KEY (“2019 Novel Coronavirus Disease” OR “2019 Novel Coronavirus Infection” OR “2019-nCoV Disease” OR “2019-nCoV Infection” OR “COVID19 Coronavirus Disease” OR “Coronavirus Disease-19” OR “SARS Coronavirus 2 Infection” OR “SARS-CoV-2 Infection” OR “Severe Acute Respiratory Syndrome Coronavirus 2 Infection” OR “Covid 19” OR “covid-19” OR “covid19” OR “coronavirus” OR “SARS-COV-2” OR “SARS-COV2” OR “COVID disease” OR “COVID sequelae” OR “COVID complications” OR “COVID survivors” OR “COVID infection” OR “COVID infections”))) AND ((INDEXTERMS (“Voice Quality”) OR INDEXTERMS (“Voice”)) OR (TITLE-ABS-KEY (“Voice Qualities” OR “Voices” OR “Voice Disorders” OR “Voice Disturbance” OR “Voice Disturbances” OR “Voice” OR “Vocal quality” OR “Voice quality” OR “vocal health” OR “voice health” OR “vocal disorder” OR “voice production” OR “voice pathology” OR “voice dysfunction” OR “vocal dysfunction”)))	20.000
Web of science	junho/2024	((TS=(“2019 Novel Coronavirus Disease” OR “2019 Novel Coronavirus Infection” OR “2019-nCoV Disease” OR “2019-nCoV Infection” OR “COVID19 Coronavirus Disease” OR “Coronavirus Disease-19” OR “SARS Coronavirus 2 Infection” OR “SARS-CoV-2 Infection” OR “Severe Acute Respiratory Syndrome Coronavirus 2 Infection” OR “Covid 19” OR “covid-19” OR “covid19” OR “coronavirus” OR “SARS-COV-2” OR “SARS-COV2” OR “COVID disease” OR “COVID sequelae” OR “COVID complications” OR “COVID survivors” OR “COVID infection” OR “COVID infections”)) AND (TS=(“Voice Qualities” OR “Voices” OR “Voice Disorders” OR “Voice Disturbance” OR “Voice Disturbances” OR “Voice” OR “Vocal quality” OR “Voice quality” OR “vocal health” OR “voice health” OR “vocal disorder” OR “voice production” OR “voice pathology” OR “voice dysfunction” OR “vocal dysfunction”)))	2858
Cochrane	junho/2024	#1 MeSH descriptor: COVID-19 #2 MeSH descriptor: Voice Quality #3 MeSH descriptor: Voice,#4:ti,ab,kw “2019 Novel Coronavirus Disease” OR “2019 Novel Coronavirus Infection” OR “2019-nCoV Disease” OR “2019-nCoV Infection” OR “COVID19 Coronavirus Disease” OR “Coronavirus Disease-19” OR “SARS Coronavirus 2 Infection” OR “SARS-CoV-2 Infection” OR “Severe Acute Respiratory Syndrome Coronavirus 2 Infection” OR “Covid 19” OR “covid-19” OR “covid19” OR “coronavirus” OR “SARS-COV-2” OR “SARS-COV2” OR “COVID disease” OR “COVID sequelae” OR “COVID complications” OR “COVID survivors” OR “COVID infection” OR “COVID infections” #5:ti,ab,kw “Voice Qualities” OR “Voices” OR “Voice Disorders” OR “Voice Disturbance” OR “Voice Disturbances” OR “Voice” OR “Vocal quality” OR “Voice quality” OR “vocal health” OR “voice health” OR “vocal disorder” OR “voice production” OR “voice pathology” OR “voice dysfunction” OR “vocal dysfunction” #6 #1 OR #2 OR #3 OR #4 #7 #5 #8 #6 AND #7	474
Literatura Cinzenta	Data	Estratégia de busca	Número de artigos
ProQuest	janeiro/2025	(“COVID-19”) AND (“Voice Quality”)	3.103
MedRxiv	janeiro/2025	(“COVID-19”) AND (“Voice Quality”)	1.349
Google Scholar	janeiro/2025	(“COVID-19”) AND (“Voice Quality”) 2019-2025	4.160

## Apêndice B Diagrama de fluxo PRISMA 2020

Fluxograma da busca e seleção de estudos relevantes.

Fonte: Dados da busca. *De*: Page et al.^(17)^.
