# Supplementary material for: ERRATUM: COVID-19-related voice disorders: a scoping review
Source: Codas. 2026 Jul 24;38(4):e20250461. doi: 10.1590/2317-1782/e20250461en (PMC13440319; doi:10.1590/2317-1782/e20250461en)
Supplement: Translation

# Translation: ERRATA: Disfunções vocais relacionadas à COVID-19: uma revisão de escopo

**DOI:** 10.1590/2317-1782/e20250461pt

Devido a erro honesto dos autores, o artigo “Disfunções vocais relacionadas à COVID-19: uma revisão de escopo” (DOI https://doi.org/10.1590/2317-1782/e20250243pt), presente em CoDAS 2026;38(3):e20250243, foi publicado sem a identificação de todos os autores.

**Onde antes se identificava:**

Aline de Souza Silva, Rodrigo Dornelas, José Roberto Lapa e Silva.

**Agora, identifica-se:**

Aline de Souza Silva, Isabella Marins Cassiano do Nascimento, Rodrigo Dornelas, José Roberto Lapa e Silva.

Os autores pedem desculpas pelo erro.

Isabella Marins Cassiano do Nascimento

**ORCID:** https://orcid.org/0009-0000-7634-5310

**Afiliação:** Faculdade de Medicina, Universidade Federal do Rio de Janeiro – UFRJ - Rio de Janeiro (RJ), Brasil.

**Contribuição:** conceituação, curadoria dos dados, investigação, metodologia, redação, rascunho original, redação.
